# Harnessing carbon potential of lignocellulosic biomass: advances in pretreatments, applications, and the transformative role of machine learning in biorefineries

**DOI:** 10.1186/s40643-025-00935-z

**Published:** 2025-09-13

**Authors:** Lakshana G. Nair, Pradeep Verma

**Affiliations:** Bioprocess and Bioenergy Laboratory (BPBEL), Department of Microbiology, Central University of Rajasthan, Bandarsindri, Kishangarh, Ajmer, Rajasthan 305817 India

**Keywords:** Lignocellulose, Pretreatment, Biorefineries, Bioadhesives, 3D printing, Nanomaterials, Biomaterials, Hydrogels, Machine learning

## Abstract

The over-exploitation of resources has depleted non-renewable energy reserves, impacting daily life. Additionally, the excessive lignocellulosic biomass (LCB) waste from agriculture and forestry is a pressing challenge. LCB is a rich carbon source that can produce renewable biofuels and help mitigate waste concerns. LCB biorefineries are essential to the circular economy, offering eco-friendly and cost-effective solutions due to low feedstock prices. LCB, an abundant source of carbon, can be employed not only to generate renewable biofuels and other valuable products but also to mitigate waste disposal problems. LCB biorefineries are at the forefront of the circular economy, providing environmentally friendly and economically viable solutions due to the lower cost of LCB feedstocks. To enhance the efficiency of biorefineries, it is essential to overcome the recalcitrance of LCB through pretreatment, which improves the feedstock characteristics. Furthermore, exploring new methodologies and generating products beyond traditional biofuel conversions has revealed a wide range of useful products with applicability across numerous sectors. This review focuses on various trends in LCB pretreatment, highlighting current advancements in the biorefinery sector and exploring the search for innovative products and applications. This includes 3D printing, activated carbon as a biosorbent, and innovations in biocomposites and bio-adhesives aimed at sustainability. In addition, the use of LCB components in biomedical applications, such as antimicrobial/antiviral compounds, hydrogels, and the potential of cello-oligosaccharides, is explored. Lastly, the integration of machine learning in biorefineries further optimizes pretreatment and processing technologies.

## Graphical Abstract

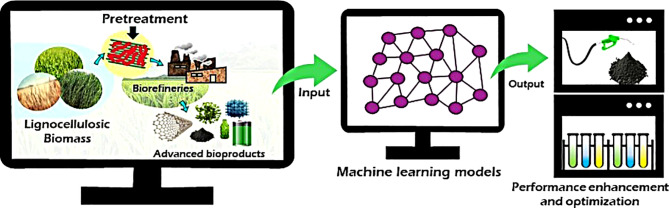

## Introduction

The cause of the population explosion has marked the rise of significant challenges in addressing various environmental issues and hindered progress in achieving the Sustainable Development Goals (SDGs). Unregulated human activities are indeed the primary source of such outcomes, ranging from environmental pollution to resource depletion and climate change. The profound dependence of mankind on fossil fuels and the unsustainable agriculture practices have resulted in the depletion and additional waste generation (Venkatramanan et al. 2021; Nair et al. 2022a). Piling up waste is hazardous to both the environment and public health and should be managed properly. Although man-made wastes form one part of the waste pile-up, biomass waste resulting from forestry and agriculture cannot be ignored altogether. The existence of large amounts of such waste causes huge risks, although they have a biodegradable nature. Moreover, biomass, particularly lignocellulosic biomass (LCB) like plant waste, is a rich powerhouse of many value-added products, ranging from biofuels, biomaterials, and platform chemicals (Mujtaba et al. 2023). Thus, the treatment of these wastes can then be an opportunity, not a problem. Urgent action is hence required to establish sustainable practices to ameliorate waste. Accordingly, the circular economy shows a promising role in the efficient utilization of biological sources for high-value-added product generation.

LCB comprises different biopolymers like lignin, cellulose, and hemicellulose, and it has major functions in biorefineries due to its ubiquitous abundance and non-competitiveness with the sources. It also acts as a potential platform for the manufacture of fuels, green chemicals, etc. (Okabe et al. 2024). LCB can also be used for the generation of biocomposites, packaging materials, drug carriers, wound dressings, biosorbents, etc. (Manyatshe et al. 2022). Agricultural crop residues, such as wheat, cotton, and rice straws, as well as husks, chaff, cobs, bagasse, leaves, and peels from other plant biomass, constitute the LCB (Haque et al. 2017). The drawback of using this biomass directly is its lower conversion rates, attributed to the recalcitrance implied by its ultrastructure, which can be solved by an efficient pretreatment step. Implementation of efficient biomass pretreatment strategies and maximal exploitation of the pretreated biomass for product generation may help to establish productive biorefineries. Moreover, new methods and practices are being discovered, and new products are being generated, other than conventional bioproducts like bioethanol or biobutanol. Several genetic algorithms and artificial intelligence modelling have been used to study global energy and environmental issues, based on the trends and impacts of the past and current world. These studies have already forecasted the consumption of oil, natural gas, and electrical energy, which leads to a rise in CO_2_ emissions, which are especially generated from the use of fossil fuels. It is noted that until 2035, fossil fuel dependency is imminent as the primary energy source unless alternative energy sources are developed (Wagle et al. 2022).

The current review highlights the recent advancements in the biorefinery sector, specifically focusing on the pretreatment methods for lignocellulosic biomass (LCB) and transformative updates in lignocellulose biorefineries, as well as the introduction of new products. The key developments covered include the 3D printing of lignocellulosic biopolymers, the use of activated carbon as a biosorbent, other innovations in nanomaterials and cellulose nanofibrils, and the emergence of bio-adhesives as alternatives to traditional chemical-based adhesives. The review also addresses new trends in biocomposites and LCB-based biomaterials aimed at sustainability, such as bioplastics and biocompatible alternatives to lignin-based materials. Additionally, it emphasizes the biomedical applications of LCB-derived products, including hydrogels and their antiviral and antimicrobial properties. Furthermore, the review discusses the status of LCB-derived carbon anodes in batteries and supercapacitors, as well as LCB-based sensors. It also explores the prebiotic potential of cello-oligosaccharides. Finally, the review examines the impact of machine learning in biorefineries, highlighting areas such as predictive modeling and real-time monitoring, which present future opportunities and strategies for improving LCB-based derivatives in biorefineries.

## Lignocellulosic biomass (LCB) complex structure and its recalcitrant nature

Lignocellulosic biomass (LCB), a surplus from agriculture and related sectors, contains an abundance of organic carbon and is also a renewable carbon-based resource that has the potential for the production of electricity, chemicals, and numerous value-added products (Sohn et al. 2022; Osman et al. 2023). The production capabilities of the LCB and related bioproducts vary between each country as they are highly dependent on the geography, biodiversity, resource availability, and technologies (Osman et al. 2023). In general, about 80–90% of the LCB is comprised of cellulose (40–50%), hemicellulose (10–30%), lignin (10–30%), and ash (up to 20%). Other extractives (1–10%) are also usually seen in the biomass (Shuai and Luterbacher 2016). Cellulose and lignin are considered to be the most abundant carbon-rich polymers available on the earth, with 44% and 63%−66% of carbon, respectively. In addition to their high carbon contents, these polymers also possess various unique properties like the 3D carbon structures, interconnected networks, high porosity, and abundant functional materials (Siddiqa et al. 2023a).

Cellulose is an unbranched polymer of β-D-glucopyranose units, interconnected by β-1,4 glycosidic bonds. It has the disaccharide cellobiose as its repeating unit (Ashokkumar et al. 2024). Cellulose, known as the most abundant polymer available on the planet (Haldar and Purkait 2021), is useful in the manufacture of films, biofuels, composites, fibers, etc. This is attributed to their qualities like biocompatibility, hydrophilicity, presence of reactive-OH groups, etc. (Jedvert and Heinze 2017). Hemicellulose is a branched polymer and constitutes different polysaccharides, including xylem, arabinoxylan, xyloglucan, galactomannan, glucuronoxylan, glucomannan, etc. They are held together by β-(1,4)- and/or β-(1,3)-glycosidic bonds (Zhou et al. 2017; Haldar and Purkait 2021). The protective boundary, aided by the lignin through the covalent linking to cellulose and hemicellulose, forms a three-dimensional network of the cross-linked polymer lignin, which contains phenyl-propane structural units that differ according to the aromatic groups (Xu and Ferdosian 2017; Baruah et al. 2018). LCB is also a storehouse of extractive minerals like magnesium, calcium, potassium, silicon, etc., which protect plants against microbial attacks and provide a distinct color and smell to the wood (Xu 2010; Milagres et al. 2011). Additionally, other components like proteins, terpenoids, resins, gums, chlorophylls, pectins, fatty acids, and different phenolic substances may also be found in the LCBs in small quantities (Kumar et al. 2020; Sharma et al. 2023).

The presence of lignin in the LCB induces resistance in the conversion of cellulose and hemicellulose fractions into the production of fermentable sugars. Hence, it needs to be removed for the exposure of cellulose and hemicellulose fractions for their fermentation into alcohols with the help of enzymes or microorganisms (Nanda et al. 2014). In the LCB cell wall, lignin functions as a natural glue to bind the hemicellulose and cellulose. The complex and inherent polymer structure of the lignin and the intermolecular linkages attributed to it culminate in the recalcitrance of the biomass, which is a drawback in LCB biorefineries (Wu et al. 2023). Thus, a pretreatment step becomes necessary for the breakdown of lignin from the other polymeric components without causing any loss of its original structure (Vaithyanathan et al. 2023).

**Fig. 1:**
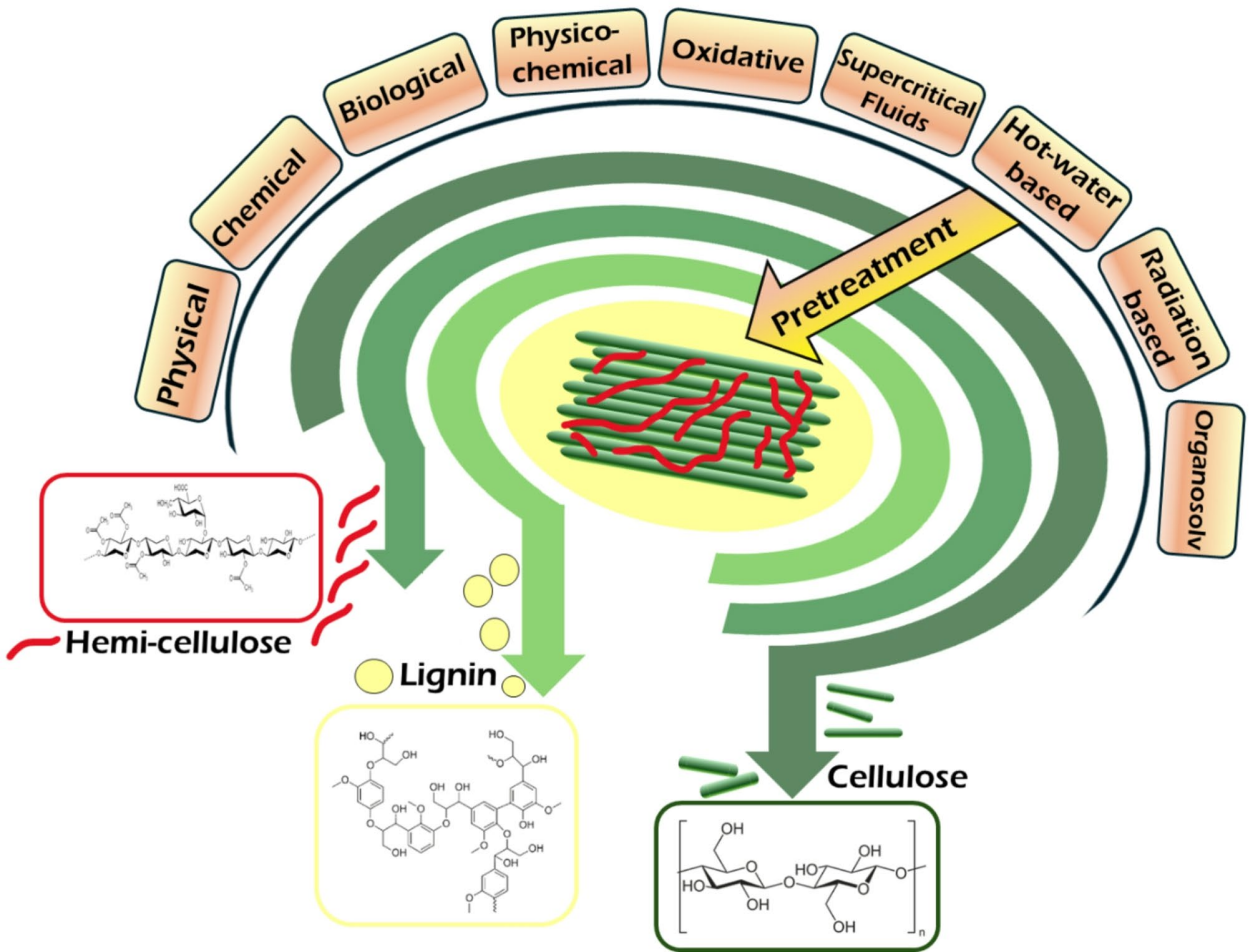
Representation of the general breakdown of lignocellulose recalcitrance using different pretreatments into its different components

LCB recalcitrance is also supported by the crystalline nature of cellulose, which makes it resistant to acids, enzymes, and swelling in water. In comparison, amorphous cellulose hydrolyzes faster in comparison to crystalline cellulose (Himmel et al. 2007; Ravindran and Jaiswal 2016; Lynd et al. 2002). The contribution of hemicellulose to the recalcitrance is limited as it only acts as a physical barrier, although the removal of hemicellulose has also aided in improved enzymatic hydrolysis rates in pretreated biomass (Nair et al. 2022a). Lignin is also known to adsorb enzymes, leading to their deactivation (Yu et al., 2012). During cellulosic conversions, cellulose plays a negative role and physically restricts the availability of polysaccharides by acting as a barrier, blocking cellulase (Nair et al. 2023). The integration of all these effects takes a toll on LCB conversion into value-added products. In this light, an efficient pretreatment strategy may be able to break the cross-links between the LCB and separate the different components. An efficient pretreatment method may not only facilitate the lignocellulosic fragmentation but also modify its structure and increase the surface area (for higher enzymatic conversions) (Jørgensen et al. 2007; Roy et al. 2020) to overcome the mass transfer barriers during the processing in biorefineries. Mass transfer effects on the LCB during chemical pretreatments have been analyzed in different studies. In general, the mass transfer barriers refer to the physical barriers that limit the movement of enzymes into and within the LCB during enzymatic hydrolysis in the biorefineries. It has been observed that at times, the residual lignin in the pretreated substrates and the cellulase interact with each other, leading to non-productive adsorptions and causing mass barrier transfer of cellulases as a result of the pore structures of LCB with lignin attached to the surface of the substrate (Zhang et al., 2025). The imposition of a mass transfer barrier often reduced the productivity of LCB biorefineries. Hence, pretreatment becomes imperative to overcome these mass transfer barriers and enhance the process of reactivity. Compared to herbaceous plants like coniferous woods, wood biomass has a compact structure and high lignification thickness, which results in extremely low pore structure, thereby increasing its mass transfer resistance. Besides the porous properties of the LCB, the different interface characteristics involved in the biorefineries, like the polarity, surface tension, contact angle, component distribution on the surface, etc., play a major role in the mass transfer mechanisms and the enzymatic reactions (Sun et al., 2023). The enzymatic digestibility decreases with an increase in particle size. Physical pretreatments like ball milling are known to mitigate the limitation of mass transfer in the enzymatic reactions, as they reduce the particle size of the biomass. Integrated mechanochemical approaches are also known to improve the mass transfer barriers (Pérez-Merchán et al., 2022). Ultrafine grinding technologies have also been known to improve the mass transfers by physically altering the structure and composition of the biomass (Zhang et al. 2016). An organosolv pretreatment assisted by a carbocation scavenger has also been reported as efficient in mitigating the surface barrier effects of lignin and improving biomass saccharification. This was assumed to be due to their lower repolymerized surface lignin production, the increased surface area, and decreased lignin coverage (Chu et al., 2021). It was observed that in the ozone pretreatment of wheat straw, the insoluble lignin in the outermost layer of the substrate reacts with ozone, leading to pore closures and forming an impermeable barrier or ‘‘cuticle’’. This prevents ozone from reaching the interior lignin, effectively shutting down mass transfer (Bhattarai et al., 2015). The mass transfer penetration involving the impregnation of the synthesized catalysts into the LCB are considered to be more complex than others as it occurs through the porous LCB structures (Sai Bharadwaj et al., 2024). The use of supercritical fluids in pretreatment are also known to permit swift mass transfers. The deep eutectic pretreatment at high temperatures also enhances the mass transfers. Ultrasound pretreatments are also known to aid in sufficient mass transfers (Mankar et al., 2021). Some of the pretreatment methods employed for the breakdown of LCB components are portrayed in Fig. 1.

## Recent advancements in LCB pretreatment methods for biomass conversion

The structural components of lignocellulosic biomass (LCB), cellulose, hemicellulose, and lignin, are intricately organized in a heterogeneous, three-dimensional matrix. The high crystallinity of cellulose, the hydrophobic character of lignin, and cellulose encapsulation within the lignin–hemicellulose framework collectively contribute to the biomass’s resistance to deconstruction (Singhvi and Gokhale 2019). Two primary factors account for this recalcitrance: (i) the densely packed structure of cellulose fibrils, which restricts enzymatic penetration, and (ii) the outer layer of lignin and hemicellulose, which acts as a barrier between enzymes and their substrate. Additionally, lignin has been shown to bind directly to the cellulose-binding modules of hydrolytic enzymes, further inhibiting their activity (Berlin et al. 2005). Studies have demonstrated that delignification can significantly enhance enzymatic hydrolysis efficiency, increasing the yield of cellulose conversion from approximately 20% to as high as 98% (Mooney et al. 1998; Petridis and Smith 2008).

Transforming biomass into renewable polymers, fuels, and chemical precursors not only reduces reliance on fossil resources but also mitigates greenhouse gas emissions (Rihko-Struckmann et al. 2020). To enable this sustainable transition, pretreatment is a critical preliminary step that addresses biomass recalcitrance. Pretreatment disrupts the molecular associations within the LCB matrix, facilitating the separation of cellulose, hemicellulose, and lignin. It also alters the biomass structure, increases its surface area, and lowers crystallinity, thereby enhancing enzymatic access during hydrolysis (Roy et al. 2020). A variety of pretreatment methods exist, including chemical, physical, biological, physicochemical, organosolv, and green solvent approaches. Given that pretreatment represents one of the costliest stages in industrial bioprocessing, it is essential to develop strategies that minimize chemical usage, reduce energy inputs, and prevent polysaccharide degradation (Nair et al. 2022b). The different pretreatment methods employed in the biorefineries have been tabulated in Table 1 and the comparison of efficiencies of different pretreatments has been portrayed in Table 2.

**Table 1:** Different pretreatment methodologies employed in the biorefinery sector

Category	Method		Mechanism	Advantages	Disadvantages	References
Physical		Milling, Grinding, Extrusion	Reduces particle size, increases surface area	Simple, increases accessibility	High energy input	(West et al. 2019; Roy et al. 2020)
Chemical		Acid, Alkali, Oxidising agents	Hydrolyzes hemicellulose/lignin, alters lignin–carbohydrate complex	High efficiency, good sugar release	Corrosive, waste disposal issues	(Sun and Cheng 2002; Loow et al. 2016)
Physiochemical		Steam Explosion, AFEX	Uses temperature, pressure, and chemicals to alter structure	Less chemical use, scalable	Degradation of sugars (esp. hemicellulose)	(Auxenfans et al. 2017; Chundawat et al. 2020)
Biological		Fungal, Bacterial	Uses microorganisms or enzymes to degrade lignin or hemicellulose	Environmentally friendly, low-energy	Slow rate, large space/time needed	(Bhatia et al. 2017; Baruah et al. 2018)
Microwave-assisted		Microwave	Rapid heating disrupts biomass structure	Fast, energy-efficient enhances chemical reactions	Expensive equipment, uneven heating at scale	(Bhardwaj et al. 2020; Nair and Verma 2025)
Ultrasound-assisted		Ultrasonication	Cavitation disrupts cell walls and increases porosity	Low energy, mild conditions, increases enzyme efficiency	Limited penetration depth, scaling issues	(Rehman et al. 2012; Kumar and Sharma 2017)
Supercritical Fluid		Supercritical CO₂, water, ethanol	High-pressure fluid breaks down lignin and hemicellulose	Non-toxic solvents, tunable selectivity, and solvent recovery	High-pressure requirement, costly	(Pasquini et al. 2005; Tomás-Pejó et al. 2011)
Deep Eutectic Solvents		Choline chloride + amides, amines, monocarboxylic acids, etc.	Solubilizes lignin via hydrogen bonding	Biodegradable, low toxicity, recyclable	Recovery challenges	(Xu et al. 2016; Satlewal et al. 2018)
Hydrothermal/Liquid Hot Water		Hot water at 160–240 °C	Autohydrolysis of hemicellulose, lignin modification	No chemicals are needed, generates fewer inhibitors	Limited delignification, energy demand	(Laskar et al. 2013; Roy et al. 2020)
Ionic Liquid		Ionic liquids	ILs disrupt crystalline cellulose	High yield, can recover ILs, and tailored solvents	IL cost, enzyme deactivation risk	(Brandt et al. 2013)
Ozonolysis		Ozone treatment at low temperature	Selective lignin degradation via oxidative cleavage	Mild, minimal cellulose damage	High ozone cost, gas handling issues	(Karimi et al. 2006; Roy et al. 2020)
Organosolv		Organic solvents at high temperatures	Increases pore volume and surface area of biomass	High lignin dissolution, solvent recovery	Lignin condensation and formation of undesirable degradation compounds	(Zhang et al. 2016; Chin et al. 2020)

**Table 2:** Efficiencies of different methods of pretreatments

Category	Method	Conditions	Biomass used	Efficiency/Result	References
Physical	Milling	3 to 7 h	Bamboo residues	39.2–53.9% lignin removal	(Yang et al., 2021)
	Extrusion	Screw speed of 150 rpm and a barrel temperature of 180 °C with a moisture content of 25%	Pine wood chips	Maximum cellulose, hemicellulose, and total sugar recoveries of 65.8, 65.6, and 66.1%, respectively	(Karunanithy et al., 2012)
Chemical	**Acid**	90 °C up to 5 h, peracetic acid	hardwood and softwood	90% delignification biomass digestibility increased by 32% and 23% for hardwood and softwood	(Kundu et al., 2021)
	Alkali	80 °C, 7% NaOH, 4 h	Rice straw	71.29% lignin removal, 88.27% total reducing sugar production	(Tsegaye et al., 2019)
Physiochemical	AFEX	High severity	Rice straw	76.0% of total theoretical maximum sugar yield	(Harun et al., 2013)
Biological	Fungal	*Ceriporiopsis subvermispora*, 60 days	Sugarcane bagasse	48% lignin loss, 47% potential glucose recovery	(Machado and Ferraz, 2017)
Microwave-assisted	Microwave	Deep eutectic solvents, 140 ◦C, 14 min, 800 W	Milled softwood mixture	90.1% delignification and 93.5% glucan retention	(Ceaser et al., 2023)
Supercritical Fluid	Supercritical CO₂	150 °C for 60 min.	Corn stover and switchgrass	Highest glucose yield of 30%	(Narayanaswamy et al. 2011)
Deep Eutectic Solvents	Choline chloride: glycerol	120 °C for 60 min.	Switchgrass	89% glucose yields	(Chen et al., 2018)
Hydrothermal/Liquid Hot Water	Hot water at 160–240 °C	200 °C, 1.45 MPa for 10 min under N_2_ gas 15	Switchgrass	77.4% glucose yields	(Hu and Ragauskas, 2011)
Ionic Liquid	Ionic liquids	1-H-3-methylmorpholinium chloride, 120 °C, 5 h	Rice straw	70.1% hydrolysis yields	(Mohammadi et al., 2019)
Ozonolysis	Ozone treatment at low temperature	80 °C, 8 h	Sugarcane straw	60 and 71% of yield in glucose and xylose conversion	(Orduña Ortega et al., 2020)
Organosolv	Ethylene glycol	80 °C, 40 min	Degraded empty fruit branch	90.4 wt% cellulose	(Chin et al., 2021)

### Physical methods for LCB structural changes to access its components

Conventional physical pretreatment approaches for LCB mainly aim to reduce particle size, moisture content, and structural crystallinity. Common techniques include drying, chipping, milling, grinding, rolling, and shredding (West et al. 2019). These methods can improve hydrolysis efficiency, promote organic compound generation, and support anaerobic digestion for biofuel production (Sinha and Pandey 2011). However, using a single physical method is often insufficient for optimal enzymatic hydrolysis/digestion. Although it can assist biofuel production, combining it with chemical methods is generally recommended (Taherzadeh and Karimi 2008). Major limitations include high operational costs and instrument corrosion (Harmsen et al., 2010; Roy et al. 2020).

Advanced physical methods include microwave and ultrasonication. Sonication, a newer technique, induces both physical and chemical changes, improving accessibility for cellulolytic microbes by rupturing cellulose and hemicellulose layers through the formation of cavitation bubbles (Kumar and Sharma 2017). While the sonication time affects pretreatment efficiency, extending the duration offers minimal added benefit in terms of delignification or sugar release (Rehman et al. 2012). Another technique, pulsed electric field (PEF), uses short high-voltage pulses (5–20 kV/cm) to create pores in the cell membranes, exposing cellulose to hydrolytic agents. PEF is energy-efficient and mechanically simple due to its lack of moving parts (Kumar et al. 2009).

### Chemical pretreatments for LCB complex deconstruction to improve component accessibility

Chemical pre-treatment involves the use of acids, alkalis, or oxidizing agents. Acid hydrolysis improves delignification and sugar accessibility by breaking down hemicellulose, precipitating lignin, and enhancing microbial digestibility (Roy et al. 2020). Common acids used include sulfuric, hydrochloric, nitric, acetic, and formic (Bensah and Mensah 2013; de Vasconcelos et al. 2013). This method can achieve high lignin removal (up to 52.48%) and hemicellulose removal (Lalak et al. 2014; Roy et al. 2020), but strong acids may generate toxic by-products and damage processing equipment (Sun and Cheng 2005).

Alkaline pre-treatment relies on solvation and saponification reactions to enhance microbial access by swelling the biomass and reducing its crystallinity (Kumar et al. 2009). Alkalis like ammonium, calcium, potassium, sodium hydroxide, and sodium sulfide are used to disrupt LCB crosslinking (Alvira et al. 2010). This method increases the density and stability of cellulose but may also lead to lignin condensation and redistribution, reducing removal efficiency (Hendriks and Zeeman 2009; Li et al. 2012).

### An updated outlook and challenges in oxidative pretreatments for LCB deconstruction

Oxidative methods, traditionally used in the pulp industry, dissolve hemicellulose and degrade lignin using peroxides or peracids (Ozmihci and Kargi 2007). These cause electrophilic reactions, side-chain scissions, and bond cleavages (Hendriks and Zeeman 2009). Ozone, a potent oxidant, converts grasses into valuable chemicals like levulinic acid, vanillin, and hydroquinone (Roy et al. 2020). However, its high consumption rate makes the process expensive. Hydrogen peroxide, although effective, is unstable in alkaline conditions and decomposes in the presence of metals like Fe and Mn, requiring careful process control (Karimi et al. 2006; Roy et al. 2020). The expensive nature of the oxidative pretreatment hinders its large-scale implementation. Additionally, the production of inhibitory compounds, such as furfural and acetic acid, causes significant hemicellulose loss, thereby reducing the sugar yield (Zhou et al. 2023b).

### Combination of physicochemical processes to enhance LCB accessibility

Physiochemical pre-treatment includes pyrolysis and torrefaction. Pyrolysis involves heating biomass at high temperatures in an oxygen-free environment to produce vapors, which are condensed into bio-oil (Roy et al. 2020). The resulting oil contains diverse organic and aqueous compounds (Sukiran et al. 2011). Its high energy demands and complex setup are notable drawbacks. Torrefaction, a more efficient alternative (30%), is carried out at 200–300 °C under nitrogen. It reduces biomass moisture, increases calorific value, and makes the material hydrophobic (Prins et al. 2006). This process also enhances aromaticity and ether bond cleavage in lignin (Neupane et al. 2015; Roy et al. 2020).

### Biological pretreatments as an eco-friendly solution

Biological pretreatment is gaining attention due to its eco-friendliness, simple reactor design, and low energy requirements. It uses ligninolytic microorganisms, mainly fungi like brown, white, and soft-rot species, to degrade lignin and hemicellulose (Sánchez 2009). White rot fungi (WRF), through laccases and peroxidases, are particularly effective (Kumar et al. 2009). While fungi take weeks or months, bacterial and enzymatic methods act within hours. WRF selectively degrades lignin over cellulose, making them ideal for efficient enzymatic hydrolysis (Shirkavand et al. 2017; Fang et al. 2018; Zabed et al. 2019). Biological methods work well for hardwoods and softwoods (Rezania et al. 2020). Combining biological with thermal or chemical treatments also enhances results (Wang et al. 2012b). For example, pairing hot water treatment with WRF on *Populus tormentosa* improved hemicellulose removal (92.33%) and doubled glucose yield (Wang et al. 2012).

### A promising process of LCB pretreatment by supercritical fluid

Supercritical fluids (SCFs), such as water and CO₂ above their critical points, exhibit liquid-like solvating power and gas-like diffusivity (Haynes 2014). CO₂, with a critical point at 31.1 °C and 7.36 MPa, is particularly suitable due to its low cost, non-toxicity, and recyclability (Gao et al. 2010; Kendall et al. 1999). When moisture is present, supercritical CO₂ forms carbonic acid, catalyzing LCB hydrolysis (Tomás-Pejó et al. 2011). Higher moisture levels correlate with better hydrolysis performance, and high pressure promotes bond cleavage between lignin and cellulose (Pasquini et al. 2005; Narayanaswamy et al. 2011).

### A mild process of hot water pretreatment for sustainability

This chemical-free, sustainable approach uses high temperature and pressure to break bonds between lignocellulosic polymers based on their thermal stabilities (Wang et al. 2012a, b). It avoids equipment corrosion and pollution, making it attractive (Laskar et al. 2013). The methods do not require any additional chemicals, which makes them suitable as a cost-efficient and low-pollution method. Also, the use of water as a solvent in this method reduces the risk of instrument damage (Nair et al. 2022a).

### Ionic liquid (IL) and deep eutectic solvent (DES) pretreatments as a green approach

Ionic Liquids (ILs) are low-melting-point salts composed of ions with strong covalent interactions, enabling them to dissolve lignin, cellulose, and hemicellulose (Vekariya 2017). Depending on the composition, ILs may target all LCB components or selectively dissolve individual ones (Hou et al. 2017). Their efficacy improves when combined with acids, microwaves, or ultrasound treatments. However, their toxicity, high viscosity, recyclability issues, and cost are limitations (Roy et al. 2020).

Deep eutectic solvent (DESs), made from non-toxic natural materials, offer advantages similar to ILs but at a lower cost and with easier preparation. Typically, they include a hydrogen bond donor (e.g., lactic acid) and acceptor (e.g., choline chloride), forming a low-melting mixture. DES pre-treatment enhances digestibility, reduces biomass recalcitrance, and supports the production of value-added products (Satlewal et al. 2018). Acidic DESs are most common, followed by polyalcohol types. Microwaves and ultrasound can further boost their performance (Roy et al. 2020).

### Organosolv method as a high lignin dissolution approach

Organosolv pretreatment employs the use of organic solvents like ethanol, methanol, acetone, ethylene glycol, etc., for the extensive removal of lignin and hemicellulose from the LCB (Alvira et al. 2010). This results in the increased pore volume and surface area of the biomass, leading to enhanced enzymatic hydrolysis and saccharification rates (Zhang et al. 2016), probably due to the increased accessibility of the cellulolytic enzymes (Chin et al. 2020). Some disadvantages of the methods include the lignin condensation and formation of undesirable degradation compounds like acetic acid, furfural, phenolic components, etc. (Chin et al. 2020). Although the recovery of solvents by distillation and reuse in the pretreatment is possible, the non-optimization of the solvent recovery procedures may pose a drawback (Borand and Karaosmanoǧlu 2018; Chin et al. 2020). The high-quality lignin obtained from this process may be used for the processing of many value-added products (Borand and Karaosmanoǧlu 2018).

### High efficiency of combination pretreatments in tackling LCB recalcitrance

The combinations of different pretreatment methods have also proven to be efficient for the fractionation of LCBs. Individual pretreatment systems often have disadvantages in certain aspects of the pretreatment, which can be overcome by combining them with another method. A study by Matsakas et al. (2019) reported a maximum delignification of 79.4% and 61% saccharification yields using a hybrid organosolv-steam pretreatment in spruce biomass (Matsakas et al., 2019). A combined alkaline-mechanical fractionation of rice straw was found to improve its enzymatic hydrolysis efficiency, with a maximum of 97.34% (Areepak et al., 2022). Further, a novel sequential ionic liquid pretreatment assisted by ultrasound irradiation and biosurfactant impregnation was found to improve the ethanol yield from Cogongrass, an invasive plant species (Goshadrou, 2021). A ferric chloride-catalyzed dimethyl sulfoxide pretreatment was studied using different additives by Wei et al. (2019). A maximum glucose yield of 90.2% was observed from pretreated corn stover after enzymatic saccharification (Wei et al., 2019). Similarly, the combination of microwave pretreatment with transition metal salt and orthophosphoric acid pretreatment systems was also observed to enhance the ethanol yields after fermentation (Kumar et al. 2020).

## Tools for the analysis of environmental and economic impacts of biorefineries

Life Cycle Analysis (LCA) and Techno-Economic Analysis (TEA) of different biorefinery processes are performed to evaluate their environmental and economic viability. LCA is a standardized methodology for the evaluation of the environmental impacts of different products across their entire life span, encompassing resource extraction (cradle), production, usage, and end-of-life disposal (grave). Globally recognized as the primary environmental assessment tool, it is widely employed by both governments and private organizations to inform decision-making. Its key strength lies in the translation of environmental impacts into high-level areas such as human health and ecosystem quality, thereby enhancing the clarity and communication of findings to stakeholders and policymakers. In recent years, LCA has been extensively applied to various chemicals, aiding in the identification of major impact hotspots and potential areas for improvement (Baaqel et al. 2020). Similarly, the TEA serves as a tool for assessing a range of economic indicators. TEA can be used to evaluate technical factors such as installation, service life, and maintenance needs, as well as estimate production costs, capital investment, and payback periods. Like LCA, TEA results can also help in the identification of economic hotspots, enable comparisons between systems, and guide decision-making. Conducting TEA early in the development of new technologies is crucial to ensure their competitiveness with existing solutions and to attract industrial interest, thereby supporting further technological advancement (Davidson et al. 2021).

The maturity of the technologies and processes employed in the biorefineries spans a wide range of technology readiness levels (TRLs), from early-stage proofs-of-concept to fully commercial operations. Regardless, comprehensive evaluations of technical, economic, and environmental factors are crucial before large-scale implementation of these biorefineries. TEA offers an effective means to assess process performance, costs, and scalability, supporting informed decision-making. Studies on biorefining systems and biomass supply chains employ methods ranging from computer-aided scale-up simulations to conventional economic metrics such as cash flow, net present value, and payback period. Findings emphasize that multi-product systems enhance economic viability, while market volatility and fluctuating raw material prices remain significant challenges (Pérez-Almada et al. 2023).

Many LCA and TEA studies mainly focus on the pretreatment step, which is the most energy-intensive step in the biorefineries. Cherubini (2010) studied the LCA of a biorefinery model that produces bioethanol, bioenergy, and biochemicals from two agricultural residues, which were corn stover and wheat straw, comparing it with fossil-based reference systems delivering equivalent products and services. Results indicated that utilizing crop residues in a biorefinery could positively cut greenhouse gas (GHG) emissions by roughly 50% and reduce non-renewable energy consumption by over 80%. However, the effects of the land-use change significantly impact the GHG balance by accounting for about half of it. It was observed that in other environmental categories, such as eutrophication potential, the biorefinery systems may exceed fossil-based systems. Overall, it was concluded that the residue-based biorefinery offers a dual benefit, i.e., provides a productive outlet for surplus lignocellulosic residues and also delivers substantial environmental advantages by reducing the dependence on non-renewable energy sources (Cherubini 2010). A comparative case study of four solvents (LCA) used in the LCB pretreatment revealed that the total monetary costs of the external production may be more than double the actual cost estimated by the conventional economic assessment methods. The comparison by using fossil-derived solvents (3) and renewably sourced solvents (1) shows that the latter consumed more energy than the fossil-derived ones. This shows that the use of renewable resources does not necessarily present expected lower externalities than the fossil-derived solvents, including ionic liquids (Baaqel et al. 2020). Comparative TEA analysis has also been studied for different pretreatment methods in biorefineries, including steam explosion, dilute sulfuric acid, ammonia fibre explosion, and biological methods. The estimated sugar production costs ($/kg) for steam explosion, dilute sulfuric acid, ammonia fibre explosion, and biological pretreatments were 0.43, 0.42, 0.65, and 1.41, respectively. These findings indicate that, under the pretext of present technological conditions, steam explosion and sulfuric acid pretreatment are promising options, while the other methods require further research and development to become competitive (Baral and Shah 2017). Although individual studies on TEA and LCA are efficient in their own aspects, an integrated Environmental and Techno-Economic Assessment (ETEA) approach has emerged as a strong framework for decision-making, enabling developers and policymakers to promote the advancement of environmentally sustainable technologies. The ETEA evaluations have thus become a valuable support tool, emphasizing the significance of combining both techno-economic and environmental analysis as a critical aid in decision-making in the biorefinery sectors (Pérez-Almada et al. 2023).

Although agro-industrial waste such as LCB is plentifully available, the use of food crops is still relevant in biomass biorefineries. Hence, it becomes detrimental to analyze their impact on the environment using different parameters. In this light, the Water-Energy-Food nexus framework advocates for cross-sectoral integration and interdependence as a crucial strategy to secure resources amid the global challenges of climate change, resource scarcity, and rising, competing demands for water, energy, and food (Lazaro et al. 2022). The adoption of a Water-Energy-Food nexus in the LCA allows the analysis of their interrelationships, an indicator that measures the amount of food that could be produced, causing the same land use impact in the form of biodiversity damage as 1 MJ of the energy product, as was designed by Kock et al. (2024). The Food-Energy-Water-Waste nexus describes the interlinks between the processes of production, distribution, and consumption of the four elements. The allocation or production of food energy or water often involves the generation, distribution, and consumption of the other two factors. Unused food and related commodities often end up as waste, and their management requires the input of both energy and water. This integral interconnection underscores the value of the nexus approach as a framework for addressing sustainability challenges that arise during the individual management of each element (Feng et al. 2020). A relationship study on the water, energy, land, and food nexus for the bioethanol productivities of two small-scale bioethanol-producing factories in Nigeria (year 2020/2021) was performed by Gazal et al. (2024) by comparing it with the commercial bioethanol producer of South-Centre Brazil. The results reveal that the cultivation step was the most energy-intensive (90%), with 88% water consumption. Energy consumption can be minimized by substituting chemical fertilizers with bio-based ones and improving the efficiency of the machinery involved (Gazal et al. 2024). A Food-Energy-Water-Waste nexus case study of the Hunter region, in Australia, reflected the supply-and-demand dynamics that are typical of developed nations. Multiple scenarios, including the business-as-usual, water and wastewater management, power plant decommissioning, waste-to-energy pathways, and policy interventions, were developed to assess the nexus from both individual element and whole-system perspectives. Results highlight the advantages of generating biogas and syngas via anaerobic digestion and gasification within waste-to-energy strategies, alongside key findings for the water and energy sectors. The analysis provides a foundation for regional planning, equipping stakeholders with modeling tools to evaluate different scenarios, foster collaborative learning, and build consensus based on diverse performance indicators, which include both financial and environmental metrics (Feng et al. 2020).

## Transformative updates in lignocellulose biorefineries and the advent of new products

**Fig. 2:**
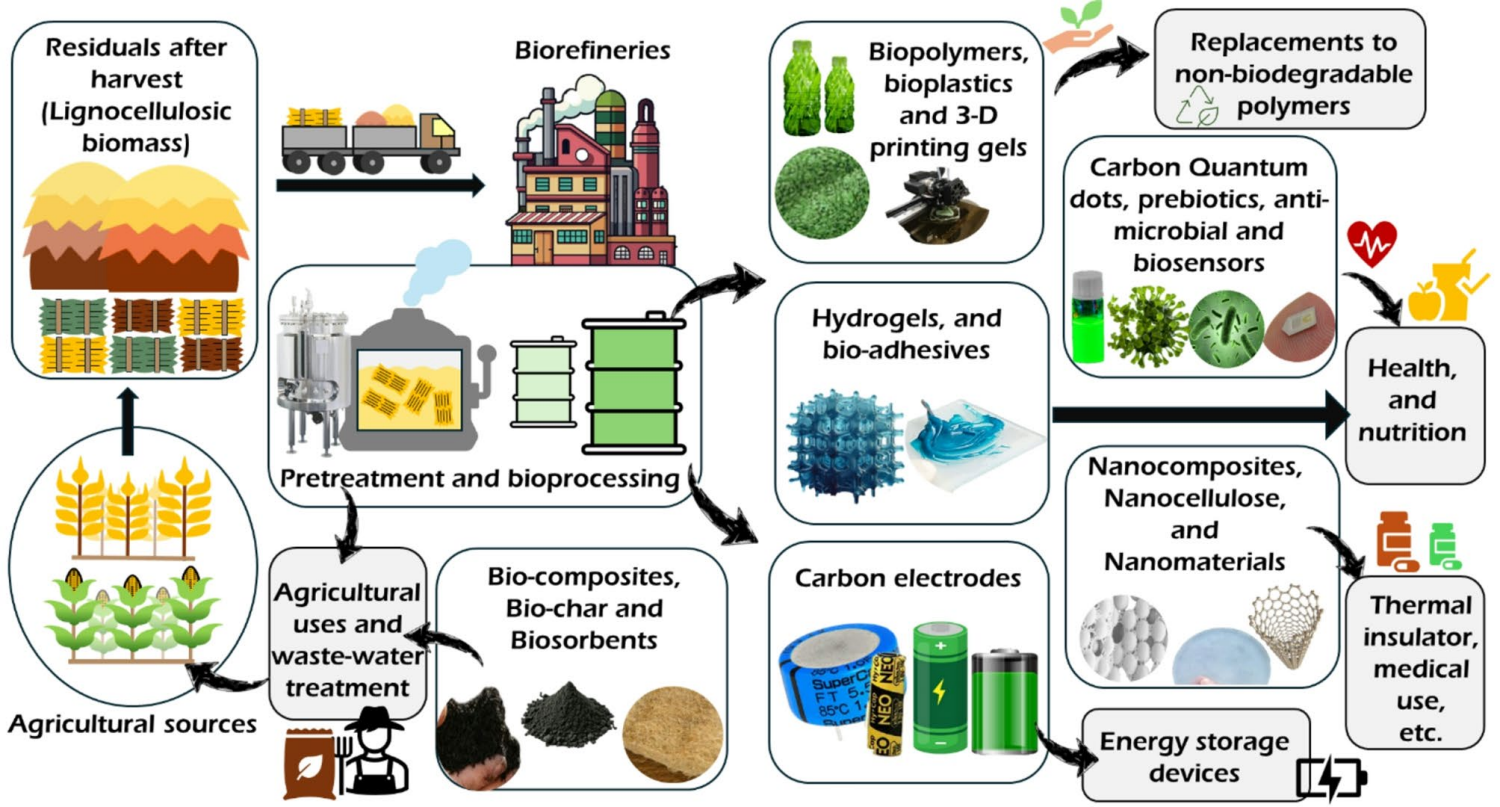
Integrative diagram of the biorefinery pathway for the new bioproducts and their uses

The LCB-based biorefineries are already well known for the production of biofuels and biomaterials. Various types of LCB hold the potential for the production of biofuels, e.g., biogas, biohydrogen, biodiesel, bioethanol, etc. LCB-based biofuels implement a carbon-negative energy approach that has been widely studied, which combines energy production with zero net carbon emissions. In this case, instead of adding carbon dioxide into the atmosphere, it is removed to accomplish a carbon-negative economy (Osman et al. 2023). Although liquid biofuels are used as engine fuels, they can also be used in other platforms such as fuel cells, engines, etc. (Demirbas 2008). The liquid biofuels replacing the fossil-derived petroleum are known to be carbon-neutral alternatives as the combustible carbon generated is balanced by its photosynthetic capture (Marriott et al. 2016). The gaseous form of biofuels, namely the biohydrogen and biomethane, also have considerable advantages as a clean energy substitute in tackling the pollution scales on a global level (Meher Kotay and Das 2008; Nair et al. 2022b). Recent research has unveiled an array of new products and applications of LCB biorefineries that are different from traditional biofuel production (Table 3). The following sections discuss the new trends and applications observed in the LCB biorefineries. Some of the recent advancements in the LCB biorefineries are portrayed in Fig. 2.

**Table 3:** Advancements in the sector of value-added production from biorefineries

Value-added product	Biomass	Type of pretreatment / Method of production	Useful part	Uses	References
3D polymer	*Cynara cardunculus* L.	Microwave-assisted organosolv fractionation using an	Cellulose-rich pulp and technical lignin	Biomedical application, biocomposites	(Cavalaglio and Gelosia 2024)
	Corn cob	Water or NaOH	Hemicellulose-rich paste	Scaffolds in tissue engineering, drug delivery, etc.	(Bahçegül et al. 2020)
Activated carbons	Oak wood	Chemical activation	Cellulose, hemicellulose, lignin	Biosorbents	(Bag et al. 2020)
	Banana peels				(Bakar et al. 2023)
	Mango seed				(Dzigbor and Chimphango 2019)
Antiviral/Antimicrobial activities	Beech wood	Microwave acidolysis	Lignin − carbohydrate complexes	Antiviral	(Li et al. 2021b)
	Japanese cedar wood	Microwave solvolysis	Lignin	Anti-bacterial activity against *Streptococcus pneumoniae*	(Okabe et al. 2023)
	Woody biomass		Lignin	Anti-SARS-CoV-2	(Okabe et al. 2024)
	Corn stover	Low-moisture anhydrous ammonia pretreatment followed by enzymatic hydrolysis	Lignin extracts	Antimicrobial activities against *Listeria innocua* and high absorbance for oxygen radicals	(Guo et al. 2018).
Carbon quantum dots	Oil palm biomass	hydrothermal conversion	Blue luminescent CQDs	Biomedical, water purification, energy storage, etc.	(Mahat and Shamsudin 2020)
	Moso bamboo	Organosolv	Organosolv lignin into carbon quantum dots	Specific fluorescence quenching in Fe^3+^; useful as nanoprobes	(Ahmad Farid et al. 2024).
	Birchwood	Coupling hydrogenolysis and hydrothermal treatment	CDQS with excellent selectivity and detection levels for different heavy metal ions, especially Fe^3+^	Great potential as fluorescent sensors for environmental monitoring	(Chen et al. 2021c).
Biocomposites	Sugarcane bagasse, peanut shell powder, and corn stover	Deep-eutectic solvent (DES)	DES-extracted cellulose-rich fibers to make bio-composite boards	Higher strength and modulus compared to the boards made with untreated fibers	(Fatima Haq et al. 2022)
	Coffee wastes	Alkaline pretreatment (NaOH)	Epoxy composites	Composite material	(Özmeral et al. 2023)
	Oil palm biomass	Ionic liquid pretreatment	Green composite boards	Superior strength and modulus	(Mahmood et al. 2016)
Bioplastics	Corn cob	Alkaline pretreatment without the bleaching process	Hydrogels to bioplastic conversion	Transparent and elastic bioplastics	(Kongklieng et al. 2024).
	Sugarcane bagasse	Acid pretreatment	Enzymatic hydrolysis using microbial co-culture	Polyhydroxyalkanoate production	(Saratale et al. 2022).
	Grass biomass	Mild alkaline hydrolysis	Functional bioplastics with anti-microbial and anti-oxidant activities	Packaging materials	(Estrada-Sotomayor et al. 2025).
Oligosaccharides	Sugarcane straw	Steam explosion pretreatment	XOS, fermentable sugars, and bioenergy	Prebiotic potential	(Brenelli et al. 2022)
	Apple bagasse	High-pressure homogenization and enzymatic hydrolysis	Pectin cello-oligo and cello-oligosaccharides		(Manthei et al. 2024).
	Orange peel				
Biochar	Henequen plant (Trunk and flower stalk)	Pyrolysis	Biochar	Removal of endocrine disruptors	(Canché-Escamilla et al. 2022; Kumar et al. 2024)
	Corn stover	Microwave-assisted	Biochar has multiple uses and good catalytic efficiency	A catalyst for the co-pyrolysis of Douglas fir and low-density polyethylene	(Zou et al. 2022).
	Oat hull	Microwave-assisted	Catalyst	Conversion of waste cooking oil into biodiesel	(González et al. 2017)

### 3D printing of lignocellulosic biopolymers

3D printing (additive manufacturing) is a computer-aided technology that is used in the manufacture of materials by stacking layer on layer to finally form a monolithic 3D structure (Li et al. 2023b). The abundance of lignocellulose-derived biopolymers, in addition to their renewability and sustainability, makes them a perfect fit for 3D printing. LCB components, cellulose, hemicellulose, and lignin are all suitable for the production of bio-based raw materials for 3D printing technology (Li et al. 2023b). Typically, this technology minimizes the consumption of raw materials with additional advantages like saving energy, cost of production, etc. Moreover, the flexibility in the design and execution of products with different shapes and structures is an added benefit. 3D printing has been applied successfully in many fields, like biomedical, construction sectors, electronics, automotives, textiles, food, aerospace, etc. (Li et al. 2023b).

Each component from the LCB structure can be extracted in the biorefinery concept and could be tailored into versatile 3D printing materials for various applications. Using these individual components not only increases the sustainability of the system but also negates the side effects associated with the synthetic polymers in the case of their biomedical applications, like harmful breakdown products, degradability, decreased cell attachment, etc. (Liu et al. 2019). However, these LCB-based polymers should be prepared to be suitable for the process. Usually, biopolymers for 3D printing are modified by incorporating additives or nanoparticles or, rather, blending them with already 3D printable synthetic biopolymers (Bahcegul et al. 2022). Cellulose biopolymers are already known for their use in 3D printing. The hemicellulosic part of the lignocellulose could also be used for 3D printing, although the study in this area is limited. Xylans and mannans from the hemicellulose components can be blended with other polymers to enable them for 3D printing (Bahcegul et al. 2022). Hemicellulose pastes prepared from lignocellulose wastes like corncob were used for 3D printing without any chemical modifications by Bahçegül et al. (2020). These 3D printed biopolymers from LCBs can be employed for biomedical applications like drug delivery systems, production of nano-cellulose, wound dressings, tissue engineering, etc. (Bahçegül et al. 2020).

As cellulose already has wide applications in different industries, it is imminent that the other polymers in LCB, like hemicellulose and lignin, will be used as biomaterials for 3D printing. In this context, Bahcegul et al. (2022) reported that the alkaline soluble corn cob portions, in their crude form, without any further modifications or purification processes, can be used for 3D printing at a 45 °C printing temperature, and water content of 83%, followed by permanent fixing using ethanol. It has been reported that the basis of the 3D printing biomaterial produced in such a way is a thermos reversible-cold cold-setting gel (the alkaline soluble phase) formed by the partial evaporation of its inherent water contents (Bahcegul et al. 2022). Similarly, corn cob was also reported to have been used for the synthesis of hemicellulosic paste for 3D printing. The extracted hemicellulose fractions were either mixed with water or NaOH for the preparation of a hemicellulose paste fit for 3D printing. The scaffolds printed by using such hemicellulosic pastes are useful in the biomedical fields for the construction of scaffolds in tissue engineering, drug delivery, etc. (Bahçegül et al. 2020). In a study by Cavalaglio and Gelosia (2024), *Cynara cardunculus* L. biomass was pretreated using microwave-assisted organosolv fractionation by making use of an acidified GVL/water solution. As a result, it produced a cellulose-rich pulp and technical lignin, which was further converted into nanocrystalline cellulose (NCC), poly lactic acid (PLA), and finally, the bio-composite incorporating NCC, PLA, and lignin, which is useful for 3D printing, with potential uses in the biomedical sector. Biocomposites for 3D printing were prepared by the uniform dispersion of CNCs and technical lignin in the PLA (Cavalaglio and Gelosia 2024). Tomato-agricultural waste-based PLA has also been reported to have been used for the design of bio-based composites for 3D printing (Pemas et al. 2024).

Nanofibril aerogels (CNFs), designed from the customization of LCB, i.e., unbleached poplar pulp, have also been reported to have been used in 3D printing of thermal insulators. It was also observed to exhibit lower stresses than the conventional cellulose nanofibrils. These CNF-based aerogels are recognized as efficient thermal insulators owing to their sustainable nature, high porosity, large specific surface area, biodegradability, low thermal conductivity, and low density. Furthermore, the lignocellulose nanofibrils produced in such a way also show superior mechanical protection, moisture tolerance, thermal insulation, and UV resistance, which can be employed in the design of batteries, chips, etc., with temperature sensitivity requirements (Liu et al. 2023).

Polylactic acid (PLA) is one of the main biodegradable polymers used in 3D printing. Other than that, Polyvinyl alcohol (PVA) and Polycaprolactone (PCL) are also used in 3D printing. Generally, in extrusion-based printing, additives like natural fibers, carbon fibers, polymer fibers, hydroxyapatite, zirconium oxide, etc., are used for the improvement of printable materials’ properties (Bahçegül et al. 2020). Zhang et al. (2022) prepared an LCB-based nanofiber and a composite of PLA with internal lignin for the preparation of 3D printable filaments with enhanced performance. It was observed that CNF addition to the PLA component increased its flexural strength, and the internal lignin available in the composite showed adhesive properties, which further increased the interfacial compatibility of the composite (Zhang et al. 2022). Studies have also been focused on the improvement of the 3D printing devices for LCB-based biopolymers to enhance the printing quality, such as novel designs of the 3D printing nozzles for extrusion methods (Sharma et al. 2021). Nanocellulose-based hydrogels derived from lignocellulosic biomass are also known to have applications in the food 3D printing sector (Ma et al. 2022).

### Exploration of activated carbons from LCB as biosorbents

Biosorbents are decontaminants that can be used to clean pollutants from sources like wastewater (Asemave et al. 2021). LCB can be converted to biosorbents using chemical, thermochemical, or biological processes. In addition to charcoal and biochar, activated carbon (AC) is a pyrogenic carbonaceous material and can be derived from biomass (Asemave et al. 2021). Activated carbons (ACs) can be synthesized by using both fossil fuels and renewable materials. ACs are amorphous materials with high porosity and surface areas greater than 500 m^2^/g. Its unique properties enable its application in a variety of fields like the purification of gas and liquids, healthcare and medicine, catalysis, etc. (Bag et al. 2020). ACs can be synthesized from high-carbon-containing materials (Bakar et al. 2023). Due to their abundance, purity, and lower costs, the LCBs are considered to be an excellent choice in the generation of ACs. (Bag et al. 2020; Balotin et al. 2023). Additionally, they have low inorganic content and high carbon content, and can be employed as a sorbent in wastewater treatments, as well as other emissions (Asemave et al. 2021). The use of LCB is considered a benchmark in the production of AC, due to its environmentally safe disposal (Bery et al. 2022). Boundzanga et al. (2022) revealed that the lignin and hemicellulose fractions are the major components in the conversion of LCB into ACs. In their study, it was also observed that different agro-industrial residues (LCBs), including olive pomace, poplar wood, tomato plants, and *Miscanthus X giganteus*, could be converted into ACs by chemical activation (Boundzanga et al. 2022).

The two main processes employed for the manufacture of biobased ACs include physical and chemical activation. In the physical activation, the LCBs are converted into char form in inert conditions, followed by activation at elevated temperatures, either using steam or carbon dioxide. In the case of chemical activation, initially, the organic material gets impregnated with an activating agent and is further subjected to carbonization under inert conditions (Bag et al. 2020). The chemical activation process is more advantageous in AC production due to its lower energy and operation costs and single activation steps. This is due to their requirements of lower activation temperatures, simultaneous carbonization and activation, short activation times, and higher yields. Additionally, they also produce AC with larger surface areas and well-developed microporosity (Bakar et al. 2023).

Bag et al. (2020) studied the conversion of oak wood into activated carbons using chemical activation with KOH, and Bakar et al. (2023) reported the AC synthesis by the conversion of banana peels using phosphoric acid at 470 °C. Balotin et al. (2023) also reported the conversion of the LCB waste from *Mauritia flexuosa* L. Dzigbor and Chimphango (2019) produced a granular AC from mango seed using chemical activation with NaCl by soaking it for 5.8 h at 500 °C. Bery et al. (2022) used different LCBs, like sugarcane bagasse and sawdust, for the production of ACs. There have also been reports about the use of biomass-derived activated carbons as inhibitor adsorbents during LCB pretreatments. Freitas et al. (2019) reported that the agro-industrial residue-based AC, i.e., coconut shell-AC, showed efficient adsorption of the phenolic and furaldehyde compounds and also showed high selectivity, wherein the sugars were not adsorbed. Similarly, Preechakun et al. (2024) also reported the detoxification of the hemicellulose-rich hydrolysate from the hydrothermal pretreatment of sugarcane bagasse by using activated carbon and microporous adsorption resin. Other than these, other biosorbents include biochar and charcoal, which are used in the removal of organic, inorganic, pesticides, and other toxins from aqueous systems. They also have applicability in a variety of fields, like the manufacture of green packaging materials, electrodes for microbial fuel cells, catalysts, carbon sequestration, energy storage devices, sol-remediation agents, etc. (Asemave et al. 2021). Technical lignins, a residue from the 2-G ethanol biorefineries, are also considered a favorable alternative to produce ACs with high-performance abilities (Gomez et al. 2024).

### Antiviral/Antimicrobial activities of LCB-derived value-added products

Biomass-derived bioactive components, such as polysaccharides, proteins, phenolic compounds, and antioxidants, help inhibit bacterial growth and fungal infections, as well as reduce viral transmission (Chen et al. 2024), thereby reducing biomass waste pile-ups. The synthetic and metal-chemical materials used in biomedical approaches are rather problematic and are associated with their lower biocompatibility and non-biodegradability. Biomass-derived antimicrobial materials, in this light, become advantageous on account of their sustainable, biodegradable, as well as highly biocompatible nature (Chen et al. 2024). Additionally, the mitigation of LCB wastes and the production of such valuable products go hand in hand, increasing the sustainability of the process. The action mechanism of the antimicrobial agents is usually protein synthesis inhibition, nucleic acid inhibition, cell disruption, etc. Disruption of the cell wall may lead to increased membrane permeability, further leading to the leakage of the internal constituents of the cell. Additionally, they also suppress DNA and RNA replication. The antimicrobial components can also suppress protein synthesis by targeting the protein folding or the ribosomal subunits, thereby inhibiting their active role (Lobo et al. 2021). Chitosan, cellulose, phenols, lignins, halogenated compounds, etc., belong to the category of organic antimicrobial agents (Lobo et al. 2021). In this light, the potential of cellulose and lignin-rich LCBs is evident for use in antimicrobial approaches. The antiviral property of lignin derived from the microwave glycerolysis of sugarcane bagasse was observed to have activities against enveloped viruses as well as non-enveloped cardio viruses (Kimura et al. 2022). Microwave acidolysis-derived lignin carbohydrate complexes from beech wood were also observed to have antiviral properties. Notably, the carbohydrate moiety played a critical role in the antiviral property of the complex, and its cleavage led to a decreased antiviral activity (Li et al. 2021b). The microwave solvolysis lignin from woody biomass (Japanese cedar wood) was also observed to show anti-bacterial activity against multidrug-resistant bacteria *Streptococcus pneumoniae* (Okabe et al. 2023). Anti-SARS-CoV-2 activity was also reported from microwave solvolysis lignin from woody biomass (Okabe et al. 2024).

Nano-biomaterials synthesized from raw *Citrus limetta* peels have also been found to show antiviral activity against chikungunya virus (Choudhary et al. 2020). A study by Guo et al. (2018) employed a low-moisture anhydrous ammonia-based pretreatment followed by enzymatic hydrolysis and lignin extraction from corn stover. The lignin extracts also showed considerable antimicrobial activities against antioxidants with high oxygen radical absorbance capacity. The extracts displayed significant antimicrobial activities against *Listeria innocua* and high absorbance for oxygen radicals (Guo et al. 2018). The anti-bacterial activity portrayed by lignin is attributed to the elevated oxidative stress and rupture of the bacterial membranes. When the polyphenol structures of lignin encounter the bacterial surface, they cleave and penetrate the cell wall, which eventually ruptures the bacterial components (Li et al. 2023a).

LCB is also an efficient carbohydrate source for the production of glycoconjugates for a variety of functions. The complex carbohydrates derived from LCB, mainly the oligosaccharides and the monosaccharides, could be used in the production of glycoconjugates (Rodrigues Reis et al. 2023). It has been observed by Sarrouh et al. (2021) that the lignin found from the pretreatment hydrolysates of the coffee husk (alkaline, using NaOH) contains phenolics, which are bioactive components and can be used to reduce the occurrence of degenerative diseases like diabetes and cancer (Sarrouh et al. 2021). There have also been reports that the fruit shrubs’ twigs could be used for the synthesis of oligomeric polyphenolic compounds, which are known for their antiviral as well as antifungal potentials (Janceva and Andersone 2024).

### A state-of-the-art nanomaterials and nanocomposites

The use of lignin and hemicellulose components from the LCB has been garnering great interest from material scientists. The metal nanoparticles combined with polymer-like lactic acids become good reinforcing materials to enhance the barrier properties of the biomass materials (Dey et al. 2022). Cellulose-based aerogels can be used as nano-biomaterials after peracetic acid pretreatment of wood fibers (Lee et al. 2015). The various biopolymers that can be converted from LCB also include crystalline nanocellulose (CNC), Cellulose nanofibers (CNF), cellulose nanofibers, nanocrystalline cellulose (NCC), bio-nanocomposite, nanocomposite, lignin-containing cellulose nanofiber (LCNF), nanocarbon (ANC), lignin nanosphere (LNS), etc. Cellulosic fibers (CF) from different LCB feedstocks coupled with pretreatment in different forms, like nanocomposites, can be integrated with metal-oxide (MeO) microbiological agents. The CF-MeO has the potential for COVID-19 antimicrobial resistance, as per laboratory investigations (Azizan et al. 2023).

### Recent advancements in cellulose nanofibrils

Cellulose nanofibrils derived from LCB are good packaging agents as they are good oxygen barriers. Moreover, they are biodegradable and transparent (Trifol et al. 2021), which adds to their functionality and reduces waste pileup as compared to their synthetic counterparts. Some other LCBs that have been exploited for the production of composites include coconut husk, sabai grass, hemp, date palm, and forestry biomass (Merkel et al. 2014; Dhakal et al. 2018; Guna et al. 2019; Tanase-Opedal et al. 2019; Kumar and Saha 2024). Biomass-derived nanocellulose has many industrial applications. They can be used as transparent films in optical devices, films in bioplastics, flexible electronics, functional membranes, gels and foams, biomedical materials, etc. (Sugiarto et al. 2022).

### Multidimensional applications of carbon quantum Dots

Carbon quantum dots (CQDs) are nano-carbon materials with sizes below 10 nm. They’re also known to have excellent photo and chemical stability, biocompatibility, and good water dispersibility. They also display excellent photoluminescence properties (Mahat and Shamsudin 2020). CQDs possess excellent properties like low toxicity, good conductivity, unique photoelectronic, optical, and fluorescent performances, etc. This makes them efficient as alternatives to metallic quantum dots for an array of applications, including bioimaging, LED, drug delivery, chemical probes, electrocatalysis, photocatalysis, etc. (Jing et al. 2019).

Mahat and Shamsudin (2020) studied the hydrothermal conversion of oil palm biomass at 200 °C for 3 h into blue luminescent CQDs. The high luminescence optical properties of these materials can be applied to many fields, including biomedical, water purification, energy storage, etc. (Mahat and Shamsudin 2020). Organosolv lignin from Moso bamboo was reportedly effective for conversion into carbon quantum dots, with specific fluorescence quenching in Fe^3+^. This, in turn, showcases its potential applications as a fluorescent nanoprobe for the detection of metal ions (Ahmad Farid et al. 2024). In a study by Qiu et al. (2022), six different biomass samples were tested for the production of efficient Fe^3+^ detecting CQDs, which included sorghum stalk, rubber wood, sugarcane bagasse, Chinese fir, cassava stem, and poplar using hydrothermal methods. It was observed that the cassava-derived CDQs had excellent sensitivity for Fe^3+^ detection compared to other biomass (Qiu et al. 2022). In addition to portraying photoluminescence, some lignocellulose-derived CDQs are also known to have antibacterial and antioxidant activities. A study on lignocellulose-derived CDQs found them to possess 99% antibacterial activity against *E. coli* and about 90% DPPH radical scavenging activity (Lee and Ko 2025).

LCB-derived carbon particles are known to possess many applications. Porous carbon materials are used as supercapacitor electrodes. Hollow spherical carbons are also used in supercapacitors. Lithium-sulfur batteries make use of carbon nanosheets, and carbon nanospheres are used as high-capacity anodes in lithium-ion batteries. Further, carbon quantum dots are known to have been used in metal detection (Magagula et al. 2022).

Jing et al. (2019) studied a mild oxidation process for the conversion of biomass-derived carbons into CQDs, hydrochar, and carbonized biomass using NaOH/H_2_O_2_ solution. In addition to high CDQ yields compared to the traditional hydrothermal and strong-acid oxidation processes, they portrayed excellent quantum yields and uniform size with efficiency in Pb^2+^ detection (Jing et al. 2019). Chen et al. (2021b) studied the effect of coupling hydrogenolysis and hydrothermal treatment in birchwood. It was observed that the initial catalytic hydrogenolysis of the birch wood led to a high yield of monomeric phenols, and the remaining cellulose and hemicellulose-rich carbohydrate pulp was used for the preparation of CQDs using a one-step hydrothermal process. The CDQs thus prepared showed excellent selectivity and detection levels for different heavy metal ions, especially Fe^3+,^ and great potential as fluorescent sensors for environmental monitoring (Chen et al. 2021c). Atchudan et al. (2019) reported that the CQDs from *Phyllanthus acidus* are efficient label-free optical nanoprobes for applications in vivo live-cell imaging (Atchudan et al. 2019). Mild acidolysis-assisted hydrothermal carbonization of lignin has also been reported for the simultaneous production of green and blue fluorescent CDQs (Zhu et al. 2022). Lignin-based CQDs produced by supercritical catalysis and solvothermal treatment are also observed to possess high fluorescence, with applications in tumor-targeted labeling (Zhao et al. 2023). 

A combination of solvothermal and oxidation treatments was also applied to birch wood for the preparation of CDQs (Jia et al. 2024). CDQs have also been reported to be produced from pine wood (Zhao et al. 2020), pristine wood chips (Tao et al. 2022), corn straw (Mu et al. 2024), wheat straw, fine husk powder, wheat husk, etc. (Baishya et al. 2025).

### Bio-adhesives as an alternative to chemical-based adhesives

Bio-adhesives are bio-based resins, which are naturally occurring polymers. LCB sources like soy and corn proteins, starches, kraft/organosolv lignin, bio-glycerols, tannins, etc., are some of the feedstocks used for bio-adhesive production. Although the production of bio-adhesives is still in the incubation phase, their global demand has seen a significant rise due to their potential environmental as well as health benefits (Arias et al. 2020). These biobased adhesives have roles in the manufacture of wood panels in the furniture industry (Arias et al. 2021). As a good replacement for fossil-based adhesives, bio-adhesives hold value as an environmentally friendly and renewable polymer, which also reduces formaldehyde emissions in the wood industry (Arias et al. 2022). Dong et al. (2024) reported a co-production of highly concentrated fermentable sugars as well as bio-adhesives based on lignin through an enhanced enzymatic hydrolysis process in corncob residue. Not only did they achieve a maximum concentration of 187.1 g/L, but they also developed a bio-adhesive that could be used in plywood production with excellent adhesion as well as high surface bonding strengths (Dong et al. 2024). Bansode et al. (2021) also reported the synthesis of bio-based novolac-phenol-formaldehyde adhesives, with derivation from the LCB, and could be used in the wood industry (Bansode et al. 2021).

The solid-state fermentation of wheat and barley straws with *Streptomyces* species was found to be a suitable strategy for pretreatment, resulting in improved residual lignin fractions, which is an advantage for its applications as binders in adhesive formulations with polyurethanes. It also has an environmentally friendly nature (Borrero-López et al. 2021). The other uses of bio-based adhesives also include wood composites, medicinal applications, paper industries, packaging, and uses in high-temperature environments, etc. (Vamza et al. 2022). Bio-adhesives from cassava starch have also been reported (Monroy et al. 2019). LCB bio-resins can also be used as adhesives and coatings for metals and glass, which are generally considered difficult-to-adhere materials (Pizzi 2024). A study also reports the synthesis of lignin-glyoxal bio-adhesive from lignin-based sugarcane bagasse (Arrusli et al. 2025). Cottonseed protein and sawdust cellulose (Yue et al. 2022), rice production residues (Zidanes et al. 2024), etc., have also been reported to have a positive role in the development of bio-based adhesives.

### A new initiative for diverse applications of biopolymers

Biodegradable polymers are generally classified into different families. The first family includes the agro-polymers. e.g., the polysaccharides obtained by the fractionation of LCB. In the case of the second family, they constitute the polyesters obtained from LCB or genetically modified plants through fermentation, like polyhydroxyalkanoate (PHA). The third family contains polymers, which are, in turn, synthesized from the LCB monomers, like polylactic acid (PLA). The final and fourth family consists of polyesters, which are synthesized using petrochemical processes from fossil resources, e.g., polycaprolactone (PCL), polyestramide (PEA), etc. (Avérous and Le Digabel 2006). These biodegradable polymers have many roles in different industries. They include the packaging sector, cutlery, agriculture, hygiene, etc. The LCB-based fibers are known for their good mechanical and physical properties and, hence, have been widely used as biodegradable fillers (Avérous and Le Digabel 2006).

### Biocomposites: an emerging solution

Composites are materials that are prepared using a mixture of two or more components that differ in their physical and chemical properties, where the original components can retain their identities as well as distinct properties (Manyatshe et al. 2022). Wood-polymer composites (WPCs) are a special group of polymer composites and have specific properties like enhanced stiffness, relatively low density, and biodegradability, and can be designed as per the choice of a lignocellulosic filler. Moreover, the integration of lignocellulosic fillers can also reduce the material production costs. The by-products generated from the processing of various renewable raw materials like wood, crops, fruits, etc., could be applied as lignocellulosic fillers (Hejna et al. 2020). Citrus pomace has been observed as a good feedstock for the manufacture of pectin and lignocellulose fibers, which could be used in the development of bio-composites for mulching applications as an eco-sustainable and cost-effective approach in the light of a zero-waste approach (Zannini et al. 2021). A study by Hejna et al. (2020) reported the promising potential of lignocellulosic fillers from olive stones, wheat bran, and date seeds for use in PCL-based bio-composites (Hejna et al. 2020). Composites prepared by the incorporation of different LCB sources, like wheat straw, rice husk, and corn stalk, were observed to have an observable shift in the composite properties, such as the elastic modulus, creep rate, hardness, etc. Notably, the composites portrayed an outstanding mechanical performance and could be used as biodegradable packaging materials (Sulaiman et al. 2023).

Haq et al. (2022) reported the development of sustainable bio-composite panels from different agricultural residues like sugarcane bagasse, peanut shell powder, and corn stover using a deep-eutectic solvent (DES) pretreatment. The extracted cellulose-rich fibers (CRF) using the DES method, called DES-fibres, were used to make bio-composite boards, and it was found to have higher strength and modulus compared to the boards made with untreated fibers (Fatima Haq et al. 2022). Coffee wastes (LCB) have also been used for the development of epoxy composites using different pretreatments, and it was found that the composites possessing the highest tensile strength were found with the coffee waste filler treated with NaOH (alkaline) pretreatment, whereas the highest drop in thermal values was obtained for the coffee waste fillers which were pretreated with alkali, and followed by microwave (Özmeral et al. 2023). The demand for polymeric composites has seen a tremendous rise in the last two decades, as plant fibers (LCB) reinforced with polymer composites have many advantages and solutions over composites with synthetic fibers. Additionally, they possess properties like a non-toxic nature, low density and weight, efficient mechanical properties, biodegradability, low cost, and renewability (Qasim et al. 2020).

Bio-composites can be used as building materials, durable goods, non-contact food packaging, wastewater treatment, medical applications, etc. (Manyatshe et al. 2022; McNeill et al. 2024). Bio-composites can be used as antibacterial wound dressings, drug carriers, or adsorbents in wastewater treatment (Manyatshe et al. 2022). Bio-nanocomposites are a combination of two or more materials, with at least one material having dimensions in the nanometer range (1–100 nm). Bio-nanocomposites from LCB have many roles in medical fields, including regeneration and engineering of bone and cartilage, drug-delivery carriers, wound dressing systems, etc. (Fernandes et al. 2013). The cellulose-based nanocomposites are also used as films and packaging materials in the food industries, the manufacture of optical, electronic, and other light-responsive composites, printing and paper industries, pharmaceuticals, etc. (Fernandes et al. 2013).

### A new trend of LCB-based biomaterials for sustainability

Various LCB-based wastes have been explored in recent times for the production of eco-friendly, bio-degradable, sustainable, natural-based, and renewable biomaterials with both indoor and outdoor applications. Biological agents can be used as binders for the lignocellulosic fibers, which produce 100% natural and biodegradable biomaterials. This is not only an environmentally friendly approach but also a competition to the traditional method of material synthesis. These biomaterials, which are “grown and better than manufactured,” promise wide applications in material sciences (Angelova et al. 2021). Waste rose flowers and lavender straw biomass have also been used as feedstock for the development of the Mycelium bio-material using the fungi *Ganoderma resinaceum* GA1M (Angelova et al. 2021). LCB can be converted into materials that are rich in carbon and can be used as a sustainable waste management strategy. Some of these carbon-rich materials include biochar and hydrochar, which are porous and contain excellent catalytic, adsorptive, and soil-amending properties (Singh et al. 2023).

Reports also suggest the use of dry grinds of different LCBs, like wheat straw, olive mills, and brewing spent grains, as fillers in the poly(3-hydroxybutyrate-co-valerate) (PHBV) polymer, which is used for food packaging applications. It was observed that the composites from PHBV/wheat straw fibers had good performance in the packaging of respiring food materials, whereas in the case of water-sensitive products, the PHBV/olive mills composites were preferable (Berthet et al. 2015). Glycerol-based pretreatments are also known to have promising impacts on the fabrication of fibrous fillers for the production of bio-composites using wheat straw (Mahmood et al. 2021b).

Ionic liquid (IL) pretreatment on oil palm biomass was also found to be efficient for the preparation of green composite boards. The biocomposite prepared using the IL pretreated fiber has also shown superior strength and modulus in comparison with the composite board prepared with untreated biomass (Mahmood et al. 2016). Aquatic biomass, like the water hyacinth, water lettuce, duckweed, azolla, etc., has been studied for the production of biochar and hydrochar, with wide applications in the environment, agriculture, and other related applications (Singh et al. 2023). The slow pyrolysis of LCB, like the agro-industrial residues and other organic wastes, with a limited supply of oxygen at high temperatures, yields a solid carbon-rich (65–90%) biochar (Singh et al. 2023).

Lignin-based biomaterials are known to have major roles in tissue engineering, pharmaceuticals, wound dressing, biosensors, medical devices, etc. (Nan et al. 2022). Lignin, a major byproduct of biorefineries, can be further processed chemically to produce glues, carbon-based materials, adsorbents, bioplastics, etc. (Wang et al. 2021). It is also known to be an efficient anti-ultraviolet and hemostatic agent (Hachimi Alaoui et al. 2023). A study by Sulaeman et al. (2021) reported that the microwave-assisted hydrothermal treatment of cassava peel and almond hull aids in the conversion of food waste into advanced biomaterials like micro-fibrillated lignocellulose.

### Hydrogels: a polymeric material for biocompatible solutions

Hydrogels have many uses in agriculture, like carbon capture, water retention, hygiene, and other biomedical applications. The renewable biopolymer lignin can be exploited for the development of hydrogels on account of its biocompatibility, low cytotoxicity, anti-inflammatory effects, high antioxidant and anti-microbial properties, etc. Lignin-based hydrogels have also shown potential in many biomedical applications, including wound healing, 3D bioprinting, tissue engineering, and drug delivery (Hachimi Alaoui et al. 2023). Hydrogels or aerogels from LCB could also be used in flexible high-performance supercapacitors (quasi/solid-state) (Gu et al. 2021a). Lignocellulose is also known to be an efficient polymer matrix for the preparation of hydrogels, which can be further used in the fabrication of low-cost, biodegradable, eco-friendly, and neutral hydrogels. This has been used as both an electrolyte and separator, and activated carbon as electrodes can be assembled to form high-voltage and safe symmetrical supercapacitors (EDLC) (Qiu et al. 2020). NaOH pretreatments on oak have also been known to generate activated carbons with enhanced physical properties that produce superior supercapacitors. The NaOH-pretreated biomass was further pretreated with KOH for chemical activation and carbonization to produce activated carbons (Lim et al. 2024).

### Versatile bioplastics for green sustainability

Plastic materials produced from renewable or biodegradable sources like food waste, straws (LCB), etc., are known as bioplastics. In comparison to their petrochemical competitors, they not only reduce their dependence on fossil fuels but also possess an easily degradable nature and have a smaller carbon footprint (Zhou et al. 2023a). It was reported by Zhou et al. (2023) that high-performance bioplastics could be produced from xylose-extracted corncob from the xylose industry. The bioplastic produced in such a way was found to be water-stable, biodegradable, and recyclable, with exceptional mechanical properties as compared to conventional petrochemical plastics (Zhou et al. 2023a). Corn cob waste was also used in a study by Kongklieng et al. (2024) to produce transparent and elastic bioplastics by using alkaline pretreatment without the bleaching process. Initially, hydrogels were prepared using the pretreated biomass and dried to form transparent films, which had great potential in bioplastic development (Kongklieng et al. 2024). Sugarcane bagasse was used as a substrate and pretreated with acid, yielding high saccharification sugar yields using a microbial co-culture. The enzymatic hydrolysates were employed in the production of Polyhydroxyalkanoates (PHA), a raw material for bioplastic production (Saratale et al. 2022).

Lignin and arabinose-free xylan, which have been enzymatically and chemically modified from sugarcane bagasse, have been reported for the production of bioplastics (Bueno and Brienzo 2025). The hydrolysates from the pretreatment of LCB are also known to have been used in the production of PHA (Li et al. 2021a). Deep eutectic pretreatment and acetylation processes in LCB residues like sugarcane bagasse, boxboard waste, and wood pulp wastes were studied for the production of bioplastics. The boxboard waste produced the strongest bioplastic, followed by the wood pulp and sugarcane bagasse (weakest) (Chyerochana et al. 2025). Mild alkaline hydrolysis of grass biomass is also known to produce functional bioplastics with anti-microbial and anti-oxidant activities, with potential applications as packaging materials (Estrada-Sotomayor et al. 2025).

The production of bioplastics involves partial hydrolysis of hemicellulose, lignin, and pectin, which disrupts the plant cell walls and releases cellulose nanofibers and microfibers. These could be constituted into composites, where the cellulose nanofibrils are embedded in an amorphous polymer matrix. The composition of the raw material plays a major role in the properties of bioplastics (Estrada-Sotomayor et al. 2025). The lignocellulosic hydrolysates from different LCB sources like barley, miscanthus, and pine, containing furfural, Hydroxymethyl furfural, acetates, etc., are known to show positive impacts on the growth of *Ralstonia eutropha* 5119 and PHA production (Bhatia et al. 2019). PHAs are biodegradable and eco-friendly replacements for conventional fossil-based plastics (Al-Battashi et al. 2019). Pressurized hot water pretreatment is also known to aid in the production of PHAs, in addition to increasing the enhanced sugar production rates from the pretreated biomass (Yan et al. 2021).

### Electrochemical applications of LCB-derived carbons

LCB is known to be used as an alternative fuel in direct biomass fuel cells, which convert the polymers cellulose, hemicellulose, and lignin into electricity (Antolini 2021). LCB is used in microbial fuel cells (MFCs), wherein the biomass is directly converted into electrical energy through oxidation-reduction reactions. The LCB-based MFC may be used in bioremediation, biofuel production, and other industrially important value-added products, in addition to simultaneous electricity production (Shrivastava and Sharma 2022).

The carbon materials derived from LCBs are advantageous for applications in electrochemistry. The high carbon content in the LCB materials aids in the production of functional-carbon materials like carbon dots, activated carbons, graphitic carbon, disordered carbon, etc., for use in energy storage devices and pollutant treatments. LCB-derived artificial graphite has also been studied to alleviate the concern about the shortage of graphite anode materials (Jeong et al. 2025). Further, as compared to traditional metal-based catalysts, which have limited availability, durability, and high costs, using biomass-derived carbon materials provides an added advantage. The attractive properties of these carbon materials, like low cost, good electrical conductivity, mechanical stability, corrosion resistance, and availability in different physical structures, make them efficient for use in electrochemistry (Ramírez et al. 2024).

Biomass-derived carbon anodes and cathodes have been known to have uses in electric vehicle batteries. The big layer space closed voids, and the superior surface area of the LCB-derived carbon materials makes them efficient for their application as energy storage devices. Recent technologies for carbon generation from LCB, like carbon capture and synthesis, have been used as critical materials in electronics and EV batteries to replace lithium-based batteries. Different LCB sources like corn leaves, coffee grounds, and puffballs have been previously studied for their use in batteries based on lithium-ion, potassium-ion, and sodium-ion (Table 4). Biomass-derived carbon-silicon composites are also known to be used in both sodium and lithium-ion batteries (dos Reis et al. 2023). These LCB-derived carbon materials not only reduce environmental strains but also enhance the driving range as well as the life cycle of batteries (Oyebamiji et al. 2024). Lithium-ion batteries (LIBs) are commercially adapted energy storage devices with both small-scale and large-scale applications. However, the shortage of lithium and other raw materials is a bottleneck, which has led to the search for greener and cheaper battery systems like sodium-ion batteries (dos Reis et al. 2023). Exploitation of the carbon, from cellulose, hemicellulose, and lignin for the preparation of such anodes is economically viable and environmentally friendly, with zero-carbon emissions (Zhang et al. 2024). In a study by Hosseinzadeh et al. (2024), porous carbon anodes from furfural biorefinery residues were found to be useful for the production of LIBs (Hosseinzadeh et al. 2024). During furfural production from hemicellulose, a solid residue, a concentrated lignin fraction, is produced. This lignin fraction contains condensed C-C aromatic networks, polysaccharides, and additional furanic compounds and can be used for tailoring carbon porosity for use as anodes in the LIBs (Hosseinzadeh et al. 2024).

**Table 4:** Use of carbonaceous compounds from lignocellulosic biomass (LCB) in batteries and supercapacitors

Lignocellulose	Process involved	Type of carbon	Applications	Reference
Furfural biorefinery residues	Pyrolysis, KOH activation	Porous carbon	Anodes in Lithium-ion batteries	(Hosseinzadeh et al. 2024)
Cherry pits	KOH and H_3_PO_4_ activation	-		(Hernández-Rentero et al. 2020)
Walnut shells	Electrospinning	Porous carbon nanofibers		(Tao et al. 2019)
Corn residuals	Carbonization using molten salts	Porous carbon		(Li and Kamali 2023)
Corn leaves	Molten salt-assisted conversion	Carbon nanostructures	Li-ion storage	(Li and Kamali 2023)
Orange peels	H_3_PO_4_, Carbonization	Hard carbon anode	Sodium-ion batteries	(Saha et al. 2020).
Bamboo	Carbonization			(Li et al. 2025)
Wheat straw	Deep eutectic solvent fractionation	Lignin nanoparticles to porous biocarbon	High-performance supercapacitors	(Xu et al. 2024).
*Miscanthus* grass biomass	KOH pretreated	High micropore volume and surface area of carbon		(Yakaboylu et al. 2021)
Sugarcane bagasse	Electrospinning	Porous and flexible nanofibers	Supercapacitors	(Chen et al. 2021b)

A study by Li and Kamali (2023) performed a molten-salt-assisted conversion of corn residuals into biocarbon for its use in LIBs. It was found that the biocarbon thus produced contained an enhanced porous structure and minimal impurities, which led to enhanced Lithium-ion storage performance with regard to its discharge capacity, rate capability, and cycle stability (Li and Kamali 2023). Cherry pits were used in a study by Hernández-rentero et al. (2020) for the production of carbons for their use as anodes in LIBs (Hernández-Rentero et al. 2020). Porous carbon nanofibers, which are derived from waste walnut shells, were also found to be efficient as anode materials for LIBs (Tao et al. 2019). Hard carbon derived from orange peels, an LCB source, has also been studied as an anode in SIBs (Saha et al. 2020). Such anodes derived from LCB can be classified into nanocomposite carbons for surface modification of the anode, free-standing 3D carbon anodes, and carbons for hybrid anode structures for their use in microbial fuel cells (Moradian et al. 2022). Other than the general advantages like reduced costs, easy accessibility, and eco-friendly nature of using these materials, properties like improved battery performance in terms of cycling, durability, capacity, and stability add to their merit for use in batteries (LIBs). Adopting high-capacity nanocarbons, particularly activated carbons, may increase the energy density of the LIBs with their customizable and high surface areas, remarkable conductivity, optimized porosity, and surface functionalization. Lignin, with its aromatic building blocks, can efficiently support the formation of carbon networks with electrical conductivity (Hosseinzadeh et al. 2024). Yang et al. (2024) studied the variation of LCB composition ratios on the functionality of the 3D LCB-based carbon electrodes. It was observed that the biopolymer contents played a significant role in the physicochemical and electrochemical properties of the material. Notably, increasing the cellulose and lowering the hemicellulose (xylan) reduced the ohmic as well as the charge transfer resistance. It was also observed that the presence of high amounts of cellulose facilitates electron transfer, although the capacitance was found to have been reduced (Yang et al. 2024).

Carbon-based materials like graphene, carbon quantum dots, carbon nanotubes, and hetero atom heteroatom-doped carbons have wide uses as the materials for electrodes in electrolytic double-layer capacitors (EDLC) (Siddiqa et al. 2023b). Many LCB sources, like the corn stalk, palm petiole, algarroba wood, shells of macadamia, pistachio, rubber seed, durian, walnut, acorn, peanut, etc., have been used in studies as the starting material for AC production. These ACs have been garnering a lot of interest in recent years for the development of supercapacitors (SCs). SCs are also energy storage devices that, in comparison to batteries, possess many advantages, like high power density, fast energy delivery, and exceptional endurance towards cycling. They can also work over a large range of temperatures, but SC suffers a drawback of low energy density (Kanjana et al. 2021). KOH-pretreated *Miscanthus* grass biomass was found to have high micropore volume and surface area, high content of hydroxyl groups and ester groups, rich oxygen concentration, and sheet-like morphology. Tuning the pretreatment time between 12 and 16 h showed considerable variations in the properties of the ACs, leading to their efficiency for use in high-performance supercapacitors (Yakaboylu et al. 2021).

Kraft and organosolv lignins from the LCBs can be used for the preparation of carbon aerogels, which can be used as raw materials for the synthesis of supercapacitors (Yang et al. 2017). Sugarcane bagasse has also been used for the construction of porous and flexible nanofibers by electrospinning for use in supercapacitors (Chen et al. 2021b). Furthermore, LCB-based electrodes too have uses in the design and synthesis of flexible/wearable supercapacitors (Hu et al. 2021). Lignin nanoparticles derived from the deep eutectic solvent fractionation of wheat straw were also used for the production of porous biocarbon, which has further applications in high-performance supercapacitors (Xu et al. 2024).

### LCB-based sensors towards sustainable solutions

Different LCB-based bionanomaterials, such as nanocellulose, nano lignin, etc., are known to have been used as raw materials in the development of high-impact biosensors. Sensors play a great role in recognizing different stimuli and other external factors. LCB-based nanomaterials in sensors have been garnering tremendous attention in the biomedical sector due to their use in the maintenance of human health. It is known that nanocellulose materials possess excellent chemical, thermal, physical, mechanical, and other barrier properties, in addition to the fact that they are chemically inert. Currently, many cellulose-based physical and chemical sensors have been designed for use in pressure/strain sensors, proximity sensors, temperature sensors, humidity sensors, gas sensors, etc. It is also used in biosensors for recognizing levels of glucose, cholesterol, urea, etc. (Durmaz et al. 2023).

Hemicellulose also possesses multiple functionalities for biosensor applications. They are known to have been used in tissue engineering, human-machine interface, drug delivery, electronic skins (e-skins), cancer chemotherapies, hydrogels, artificial intelligence applications, conductive polymers, cancer chemotherapies, dye adsorption, biosurfactant chemistry, etc. Lignin-based physical, chemical, and biosensors have also been known (Durmaz et al. 2023). A Peroxidase-mimetic colloidal nanozyme produced by the ozonolysis treatment of coconut husk was known to be effective in biosensing the presence of H_2_O_2_ and H_2_O_2_-dependent bacteria in water samples (Gade et al. 2024).

Nanocellulose is also associated with the production of nano-generators, bioimaging, biosensing, piezo-electronics, etc. (Khan et al. 2024). Mahmood et al. (2021) reported that biomass-derived porous graphene can be used for the electrochemical sensing of dopamine, with impressive performance. The laser-induced graphene in the study was designed from completely biomass-based films composed of kraft lignin and cellulose nanofibres (Mahmood et al. 2021a). Lignin-derived graphene electrodes are known to serve as disposable electrodes in electrochemical biosensing, like the lactate biosensor (Meng et al. 2022). Innovative lignin-based biosensors are known to have uses in environmental monitoring, medical diagnostics, wearable electronics, etc. (Mukheja et al. 2024). Lignin and nano-lignin are also known to have been used in the design and production of biosensors and biomimetic sensors (Ojo 2023). Lignosulfonate derived from the sulfite pulping of the biomass is reported to have uses in sensors with sensitivities to electrical signals, with unique advantages including their stable, rapid, selective, and sensitive detection of dopamine without any interferences (Yuan et al. 2022).

### Prebiotic potentials of cello-oligosaccharides

Selectively fermented components, which cause specific changes in the composition and/or activity of the gastrointestinal microflora that benefit the host, are called prebiotics. Usually, they are oligosaccharide carbohydrates. Recently, cello-oligosaccharides (COS) have been garnering attention as potential prebiotics with low calorific values as well as additives in pharmaceuticals. COS is also known to have uses as animal feed to increase their body weight (Boudabbous et al. 2024). In biorefineries, oligosaccharides are a common byproduct during the production of platform chemicals. Agro-food residues are also a rich source of the production of oligosaccharides, particularly the xylo-oligosaccharides (XOS), cello-oligosaccharides (COS), and pecto-oligosaccharides (POS). Other abundant oligomer intermediates include arabinose, mannose, galactose, etc. All these components are known to possess excellent bioactivities, such as plant elicitors, prebiotics, food complements, etc. These compounds are also known to have an increasing impact on various industries like cosmetics, nutraceuticals, food, and pharmaceuticals. They are also known to be used in immune system boosting, cancer treatments, gut health, anti-adhesive action, etc. Various LCB sources, like banana peels, vine shoots, sugarbeet residues, etc., can be used for the production of oligomers. XOS from cellulose is also known to be an efficient prebiotic and food complement. They also have uses in cosmetic formulations as gelling agents, as well as for the treatment of diabetes. They are also known to have functions like immunomodulation and immunostimulation, and possess antioxidant activities. Lignin oligomers are, in turn, used in various coatings and material ingredients (Cano et al. 2020). The term “oligosaccharide” refers to a short chain of monosaccharides with 2–20 sugar units. Cello-oligosaccharides are straight-chain molecules made of glucose derived from cellulose. Thus, LCB, a huge source of cellulose, becomes an efficient feedstock for the preparation of COS (Chen et al. 2021a).

Steam explosion pretreatment on sugarcane straw has been observed to be efficient for the production of XOS, fermentable sugars, and bioenergy. Out of the recovered XOS, about 50% of them were also identified as xylobiose and xylotriose, which are already known as important prebiotics (Brenelli et al. 2022). Microbial-technology-based oligosaccharide production is very efficient for LCB bioconversions (Awasthi et al. 2022). The oligosaccharides are non-digestible components, and this being a valuable feature aids in their applications in functional meals or as nutraceutical products (Awasthi et al. 2022). The extraction of oligosaccharides from agro-waste or biomass can be beneficial in terms of both waste management and environmental improvement (Awasthi et al. 2022). High-pressure homogenization and enzymatic hydrolysis of apple bagasse and orange peel were found to produce pectin and cello-oligosaccharides with prebiotic potential (Manthei et al. 2024). In a study by Forsan et al. (2024), Banana pseudo-stem, leaves, and guava bagasse were used for the production of cello-oligosaccharides and xylo-oligosaccharides via a combination of chemical, mechanical, and enzymatic treatments (Forsan et al. 2024). COS from birch biomass (*Betula pendula*) was found to enhance the abundance of fecal microbiota in juvenile rainbow trout (*Oncorhynchus mykiss*). COS was also observed to have induced higher antioxidant activities in its gut and stream, and also generated innate immune responses (Singh et al. 2024).

### Biochar for multipurpose applications

The trunk and flower stalk of the henequen plant were used in a study by Canché-Escamilla et al. (2022) for its pyrolytic conversion into bio-oil and biochar (Canché-Escamilla et al. 2022). LCB-based biochar is also known for its application in the removal of endocrine disruptors from different sources, which otherwise have an adverse effect on the health of humans and other animals by affecting their endocrine system (Kumar et al. 2024). In a study by Zou et al. (2022), Corn stover biochar, produced using a microwave-assisted approach, was used as a catalyst for the co-pyrolysis of Douglas fir and low-density polyethylene (LDPE). It was observed that the addition of this corn stover-based biochar significantly increased the hydrogen in the syngas, decreased the wax yield, and contributed to the good selectivity of aromatics. The biochar was also found to have multiple usages (more than 10 reuses) with good catalytic efficiency (Zou et al. 2022).

Biochar from LCB is also known to have been used in soil amendment in saline soil, wherein it mitigates soil salinity using ion exchange or physical adsorption. This may be possible due to the unique properties of the biomass, including its high surface area and porosity, profuse functional groups, and high carbon content. By using these mechanisms, the biochar could stop salt leaching and decrease the time required to lower salt concentration to aid proper plant growth (Tan et al. 2021). Bamboo residue, corncobs, and coconut shells were also converted into biochar using ablative pyrolysis, with coconut char showing the highest release of volatiles (Khuenkaeo and Tippayawong 2020). Biochar addition to anaerobic digestion improves the abundance of microbial communities and facilitates the degradation of lignocellulosic biomass. Different biochars derived from walnut shells and straws of wheat, corn, and rice were employed in a study by Saif et al. (2021) to study their effects during the anaerobic digestion of LCB. It was observed that the biogas production was improved by the addition of the biochar, with the maximum increment by corn straw biochar (Saif et al. 2021).

Biochars and activated carbons from LCB are also known for soil amendment practices, like the reduction of insecticides present in the soil. Biochars derived from oak wood and coconut shells were known to aid in the reduction of the chlordecone from the soil. The activated carbons were found to be successful in removing up to 80% of the chlordecone availability in the environment (Ranguin et al. 2020). Biochar from oat hull can also be functionalized using microwave-assisted technology, and it can be used as a catalyst for the conversion of waste cooking oil into biodiesel (González et al. 2017). Magnetic biochar, synthesized by using Fe_3_O_4_ doping on eucalyptus biomass, was found to be efficient for the removal of methylene blue. The magnetic property of the biochar also resulted in its easy separation after use, which makes it efficient for its application for dye adsorption from wastewater (Lee and Lee 2024). Biochar also possesses functionalities as a catalyst in different chemical and biological processes, treatment of wastewater, electrode material, supercapacitor, soil remediation, amelioration, removal of organic and inorganic wastes, carbon sequestration, agrochemical removal, etc. (Shukla et al. 2021). A study demonstrated that deep eutectic solvent-based biochar production from LCB sources like the Caribbean pine, hybrid poplar, and corn stalk was efficient for the adsorption of Cr (VI). They utilized a one-pot method wherein an acidic deep eutectic solvent (p-Toluene sulfonic acid monohydrate-choline chloride) was used at a temperature of 140 °C (Zhang et al. 2021). Rice straw biochar is also known for methylene blue adsorption (Leng et al. 2015). The biochar yields from the pyrolysis of corn stalk, corn cob, and spruce wood were found to be comparable, although the differences in their compositions were found to cause variations in their biochar properties (Wang et al. 2022).

Nitrogen doping in biochar prepared from *Medulla tetrapanacis* was also found to be efficient for the adsorption of organic dyes and heavy metal ions (Li et al. 2022). Biochar can be used as an additional carbon supplement for plants by mixing it into the soil. It also acts as an adsorbent for the natural minerals or the minerals/nutrients that are added through the fertilizers, which prevents their leaching off. Also, biochars are known to be efficient alternatives for the production of briquettes and other pellets, which are known to be feedstocks for gasifiers (Muigai et al. 2021). Biochars are also known for reducing soil alkalinity, which is a result of the rich concentration of alkali metals such as Na, Mg, P, K, and Ca (Nanda et al. 2013).

## An outlook on machine learning influences in biorefineries

Blending conventional biomass research with advanced computational methods, like machine learning, indicates a paradigm shift. This blend of data science with biology may help in unlocking frontiers in the current understanding and utilization of LCB sources for the development of sustainable energy (Santhosh et al. 2024). Artificial intelligence (AI) has been one of the game-changing innovations in recent times to find optimized solutions and manage uncertainties in problems. Mostly, they are linked to finding and solving critical issues. They are also used in the environmental engineering sector to solve pollution issues, logistics, management of waste, treatment of wastewater, management of municipal solid waste, etc. AI has been used by many industries, organizations, and even at the government level as a foundational technology to enhance operational efficiency as well as improve financial success (Naveenkumar et al. 2023).

Mathematical modeling, like the response surface methodology (RSM), has long been used by scientists for the optimization of different LCB valorization methods. Although RSM has advantages in explaining the different interactions and non-linear interactions between the independent factors on the output of the experiments, a notable disadvantage lies in the assumption of a polynomial relationship between the independent variables and the output. In this context, the artificial intelligence-based non-conventional empirical modeling approaches like the artificial neural networks (ANNs) and adaptive neuro-fuzzy inference system (ANFIS) have garnered a lot of interest for the optimization of LCB valorization (Pradhan et al. 2022).

Machine learning (ML) is considered a favorable tool for modelling as well as optimizing the process parameters and can predict without prior knowledge. This makes them efficient in the application of processes like bioethanol production. An ML algorithm generally analyzes the inputs given to the system and changes the process conditions in order to improve the efficiency of the process. It has many applications in various sectors, including the petroleum, manufacturing, and chemical industries, as well as to learn about climatic conditions (Vinitha et al. 2024). By using ML, the software programs can anticipate more accurate outcomes without the need to explicitly instruct them to do so. The ML algorithms use past data as input in order to forecast new output values (Peng and Karimi 2023).

AI broadly encompasses the technologies that mimic the cognitive processes often associated with human intelligence, like learning and problem-solving. ML, in turn, focuses on the algorithms that keep on improving their performance, as they are exposed to more data. Deep learning, a subset of ML, makes use of multi-layered neural networks to learn from large datasets. The commonly applied ML methods include the Random Forest (RF), Decision Tree (DT) Classification, Naïve Bayes Classification (NBC), K-Nearest Neighbours (K-NN), Gradient-Boosted Decision Trees (GBDT), Artificial Neural Networks (ANN), Support Vector Machines (SVMs), etc. (Tušek et al. 2024). The machine learning approach in the biorefineries is observed to be more data-driven to optimize the biofuel systems, aligning with the environmental, social, and governance (ESG) standards. They also address the environmental issues and contribute to sustainable practices in the industries (Naveed et al. 2024).

**Fig. 3:**
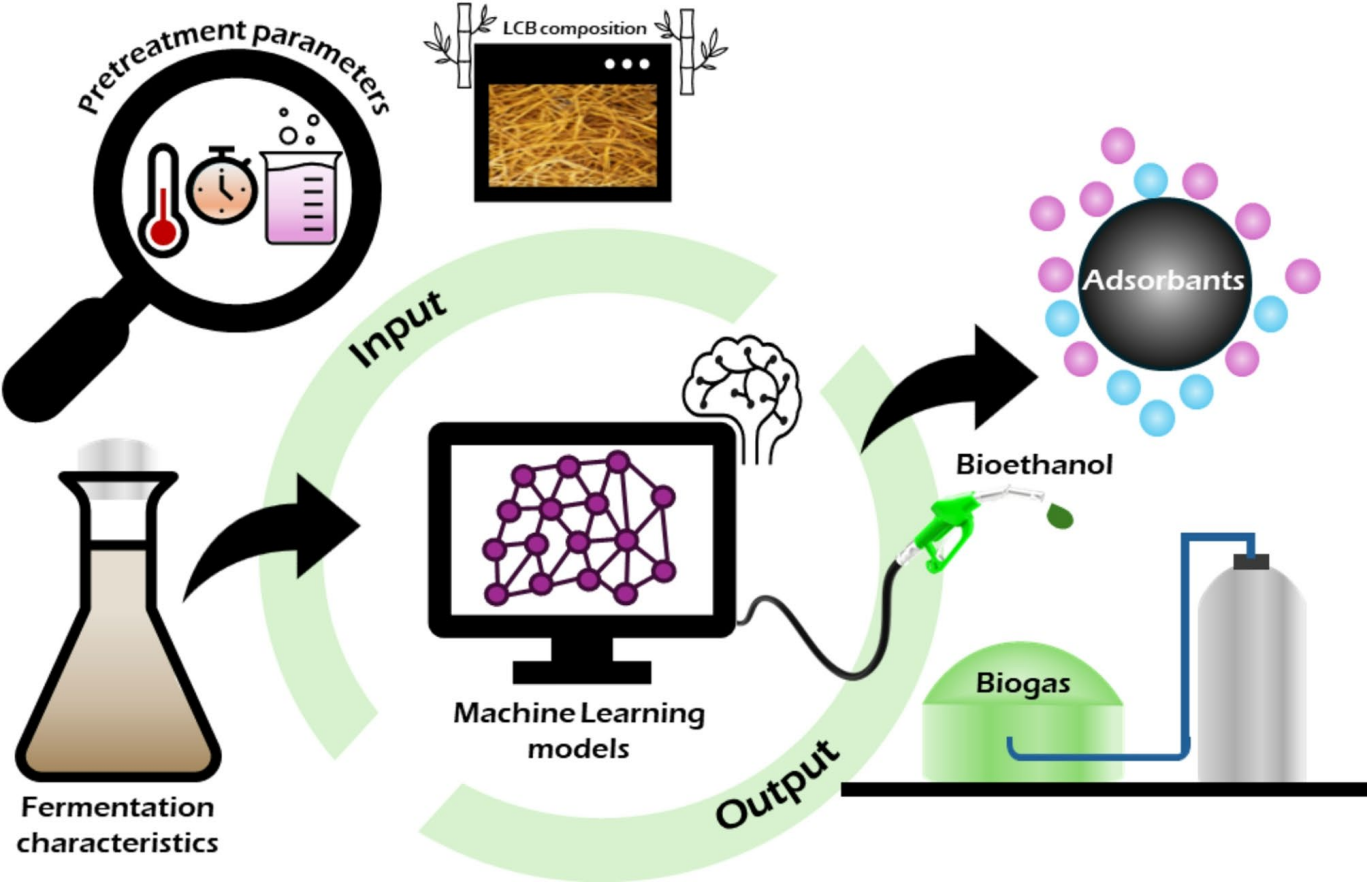
Pictorial representation of the use of Machine learning models in optimizing the biorefinery outputs

The use of AI and computer technology coupled with conventional simulation and modelling techniques may result in major benefits in the optimization of the process parameters as well as reducing the overall cost (Mateescu et al. 2022). Intelligent models have the capacity to enhance the improvement of LCB bioconversions into value-added products by making use of and determining the appropriate navigation space required for the pretreatment, as well as optimization processes. Most intelligent models focus on optimization in LCB-based research. Nonetheless, they can also be used as a predictive tool for preliminary screening and the determination of appropriate navigation spaces for the pretreatment and optimization processes (Fig. 3). The use of intelligent models for predictive modeling is mainly based on the behavior based on the available or current data, which helps determine future outcomes (Aruwajoye et al. 2022).

## Predictive modeling outcomes in biorefineries

AI and ML help in the precise modeling and prediction of the outcomes of various biofuel production methods, which allows the researchers to simulate and adjust conditions virtually before applying them in real-world scenarios. The ML algorithms have the ability to process large datasets from past production cycles to identify patterns and optimize parameters such as temperature, pH, and feedstock composition, resulting in higher yields and reduced costs. By learning from historical process data, they can forecast the impact of parameter changes on efficiency and product distribution, enabling more informed decisions. This improves current processes and also fosters innovation by aiding the development of new methods in the biorefineries. AI can also be used in the selection and genetic engineering of microorganisms and plants to enhance biofuel yields, as well as in the accurate prediction of techno-economic analysis (TEA) and life cycle assessment (LCA) outcomes for biomass-to-biofuel technologies. By leveraging these capabilities, the biofuel sector can advance sustainable, cost-effective solutions that reduce carbon emissions and promote renewable energy adoption (Okolie 2024). The involvement of AI in the biorefinery systems enhances the biowaste processing efficiency by incorporating intelligent sorting, predictive analysis for bioproduct optimization, predictive analysis, and novel valorization pathways. AI can thus be used to help in fostering environmental sustainability and help in the economic growth of the bioeconomy. (Shah et al. 2025).

To accurately monitor, control, and predict each step in the biorefineries, the integration of ML has become an interesting take, as it helps to save labor and time in the experiments. ML, a branch of AI, was developed in order to address the need for efficient tools for the analysis of large datasets due to the automation of experimental equipment and the enormous increase in computer resources, leading to the generation of numerous datasets. By making use of statistical methods, ML enables computers to learn from the data inputs and make judgments without being explicitly programmed, which also allows the systems to improve their performance by learning from experience over time. As a result, the use of ML has become a crucial factor in the development of various industries, new products and services, including biorefineries (Tušek et al. 2024).

As reviewed by Liao and Yao (2021), four areas were identified where AI has been applied in biomass biorefineries which include (i) prediction of LCB properties (ii) performance prediction of LCB bioconversions including technologies and conversion pathways (iii) prediction of the properties of bioenergy and the performance of their end-use systems and (iv) supply chain modelling and optimization. AI can be found to be invariably useful in improving the traditional models of LCB bioconversions as well as the bioenergy supply chain and optimization (Liao and Yao 2021). Integration of modelling techniques like AI has been found to be impactful in the biorefineries for the design, handling, and optimization of bioethanol production. The high accuracy and flexibility of the ANN systems are beneficial in the modelling of the different steps in bioethanol biorefineries, like the pretreatment, fermentation, and other conversion stages. The commonly used input variables in the models in the bioethanol refineries include the number of yeast cells, biomass type, fermentation time, pH, etc. As for the output parameters, they included bioethanol concentrations, reducing sugars, yields, bioethanol production, etc. (Owusu and Marfo 2023). A bibliometric analysis by Coşgun et al. in 2023 reveals that ML applications in bioethanol have been a seemingly researched topic, with biochar and biogas also getting increased attention in recent years. ANN was found to be the most used algorithm, followed by the RSM and the Random Forest. The most common output variable was also observed to be bioethanol concentration, while the fermentable sugar and glucose concentrations have also been widely studied (Coşgun et al. 2023). The use of different ML models in biorefineries has been tabulated in Table 5. Although, high-quality data is essential for developing ML models to predict biorefinery processes, collection of related data is often expensive, time-intensive, and also have inconsistencies in the literature. Therefore, a careful design of the application-specific dataset is critical, as the accuracy of the model is influenced by both the quality and quantity of the data involved. In order to address the prevailing data scarcity, ML algorithms which are suited for small datasets like the SVM, or the simulated datasets from biorefinery models (e.g. Pyrolysis; chemical conversion). While simulated data offer lower fidelity, they can also be combined with high-fidelity experimental results in a multi-fidelity modeling, improving the prediction accuracy while reducing reliance on costly experiments (Akinpelu et al. 2023).

**Table 5:** Applications of different machine learning models in biorefineries

Feedstock	Machine learning models used	Biorefinery function	Input parameters	Dataset involved	Output parameters	Correlation Coefficient (*R*^2^)	Reference
Maize stover, soybean straw and sunflower stalk	XGB	Thermal pretreatment and delignification	Pretreatment parameters-temperature, duration	Experimental data (15 runs for each crop)	Delignification- acid-insoluble detergent lignin (AIDL) content	0.756–0.980	(Kovačić et al. 2024)
Sugarcane bagasse	ANN	Alkaline hydrogen peroxide pretreatment	Temperature concentration FTIR analysis	Experimental data, FTIR analysis	Delignification rate	Near to 1	(Valim et al. 2017).
Sugarcane bagasse	ANN-PSO	An increase of 10% in the bioethanol concentration	Total soluble solids, purity, acidity, contamination, treatment time temperature, viability	Data set from the ethanol and sugar production mill in the state of São Paulo, Brazil (2015 harvest)	Wine (ethanol concentration)	0.91	(Pereira et al. 2020)
Rice straw	ANN	Microwave-assisted alkali pretreatment	NaoH concentration Microwave irradiation Time of irradiation	Experimental data (20 runs)	Cellulose recovery	0.997 for pretreatment and	(Parkhey et al. 2020)
		Saccharification of pretreated biomass	Rice straw concentration Enzyme load Hydrolysis time Tween 80 concentration	Experimental data (30 runs)	Saccharification efficiency	0.9941 for enzymatic saccharification	
Cassava peels	ANN	Soaking in dilute hydrochloric acid followed by autoclave thermal treatment	Soaking temperature Soaking time Autoclave duration Hydrochloric acid concentration Percentage solid loading	Experimental data (46 runs)	Fermentable sugar concentration	> 0.82	(Aruwajoye et al. 2022)
					Combined severity factor	> 0.93	
	DT				Fermentable sugar concentration	> 0.99	
					Combined severity factor	> 0.77	
Pretreated and untreated Rice straw samples	ANN	The difference in LCB compositions in different Rice straw samples	Augmented FTIR spectroscopic data	Data of FTIR spectra and cellulose, hemi- cellulose and lignin composition of the 18 RS were obtained from a published study	Cellulose	0.9774	(Pushpa et al. 2023)
					Hemicellulose	0.9799	
					Lignin	0.9795	
	PLSR				Cellulose	0.9904	
					Hemicellulose	0.9826	
					Lignin	0.9829	
Sago trunks	PSO–ANN	Microwave-assisted pretreatment and enzymatic hydrolysis	Microwave power Exposure time Solid loading	Experimental data (~ 17 runs)	Glucose	0.9939	(Ethaib et al. 2018)
					Xylose	0.9479	
Rice Husk	ANN	Alkali Pretreatment	Pretreatment time Solid loading NaOH concentration	Experimental data (20 runs)	Glucose	0.9898	(Nikzad et al. 2015)
					Xylose	0.9802	
Oil Palm Trunk Sap	ANFIS-PSO	Fermentation	Fermentation time pH Temperature Sugar component	Experimental data	Bioethanol concentration	0.9991	(Ezzatzadegan et al. 2021).
Corn stover	ANN	Ammonia Based Pretreatment Optimization	Residence time Ammonia loading Water loading Temperature	Experimental data (30 runs)	Glucose	0.9986	(Hundie 2021)
					Xylose	0.9987	
					Total sugar yields	0.9973	
*Arachis hypogea* Shells	ANN	Combined pretreatments by particle size reduction and Fe_3_O_4_ addition	Temperature Retention time Pretreatment methods	Experimental data	Biogas yields	0.9496	(Olatunji et al. 2023)
	FCM-ANFIS					0.9850	
Wheat straw	FFNN	Thermo-alkaline pretreatment	Chemical agent Treatment duration Number of days	Experimental data	Amount of biogas production	0.999	(Alrowais et al. 2024)
	RNNs					1.0	
	NARX					0.999	
Apple pomace	ANN	Optimization of enzyme hydrolysis	Substrate loading Enzyme loading, temperature Initial ph Combination of these parameters	Experimental data	Glucose and reducing sugars	0.99	(Gama et al. 2017)
Wheat straw	ANN	Phosphoric acid plus hydrogen peroxide (PHP) pretreatment	Time Temperature Concentration of H_3_PO_4_ Concentration of H_2_O_2_	Experimental data	Cellulose recovery	0.8070–0.9989	(Wang et al. 2024)
Alkali-treated corn stover with poultry manure	ANN	Anaerobic co-digestion	Temperature Hydraulic retention time pH Ratio of poultry manure to alkali-treated corn stover ratio	Experimental data (30 runs)	Biogas yield	0.9998	(Aklilu and Waday 2023)
Paddy straw	FANN	Dilute acid hydrolysis fermentation	Temperature pH Time	Experimental data (20 runs)	Bioethanol yield	0.966	(Jayakumar et al. 2024)
	NARX					0.869	
Cocoa pod shells	ANN	Induction-assisted in situ nanoparticle synthesis and simultaneous hydrolysis	Acid concentration Biomass load Time of induction heating Amount of fecl_3_	Experimental data (~ 31 runs)	Total sugar concentration	0.9	(Vibha et al. 2024)
					Reducing sugar concentration	0.87	
Bamboo	ANN-NSGA-II	Alkali pretreatment and enzymatic saccharification	Biomass loading Enzyme loading Surface concentration Incubation time	Experimental data	Reducing sugar yields	0.97–0.99	(Nirmal et al. 2023)
Mixed Vegetable Waste	ANN	Organic Acid Pretreatments	Time Temperature Acid concentration	Experimental data (~ 12 runs)	Reducing sugar yield	0.97	(Dharmalingam et al. 2023)
Coffee waste	ANN	Efficiency without pretreatment and with microwave and ultrasound pretreatments	Dosage Density Water absorption Bond strength Compressive strength Moisture values Fire resistance	Experimental data	Thermal conductivity values	0.99	(Yildiz and Kalem 2023)
Sugar beet shreds	ANN	Biosorbent function	Initial Cu^2+^concentration Time Particle size	Experimental data (42 sets) from their current and previous studies	Adsorption capacity for Cu^2+^	0.988	(Kukić et al. 2023)
Bamboo dust	ANN	Biogas production with cattle dung as co-substrate	Composition Temperature Time	Experimental data	Specific biogas production	0.9972–0.9991	(Ghatak and Ghatak 2018)
Sugarcane bagasse						0.9976–0.9997	
Saw dust						0.9964–0.9998	
Pretreated corn stover	ANN	Effect of enzymatic saccharification and fermentation on polyhydroxy butyrate production	Solids loading, enzyme loading, and saccharification time	Experimental data (30 runs)	Reducing sugar yield	0.94871–0.98417	(Tantoco et al. 2023)
Napier grass	BPNN	Enzymatic hydrolysis of steam-exploded napiergrass with alkaline treatment	Steam explosion parameters; Temperature Time Particle size	Experimental data	Enzymatic digestibility	0.975–0.988	(Chang et al. 2011)
	MLR					0.833–0.950	
	PLS					0.823–0.849	
Agricultural residues, forestry byproducts, and specific energy crops	GBM	Anaerobic Digestion	18 features related to biomass characteristics and reactor conditions	Dataset with 18 features from primary and secondary sources	Specific methane yield	0.93	(Santhosh et al. 2024)
Non-edible seed cake	ANN	Autoclave-assisted HCl hydrolysis	Time of hydrolysis HCL concentration	Experimental data (~ 12 runs)	Reducing sugar concentration	0.975	(Shet et al. 2018)
Palm oil mill effluent	ANN-PSO	Co-digestion with cattle manure	Palm oil mill effluent concentration Cattle manure Concentration H_2_O_2_ concentration Ammonium bicarbonate concentration	Experimental data	Biogas yield	0.9923	(Zaied et al. 2023)

Onu et al. (2024) performed illustrative case studies on three different aspects of the biorefinery and machine learning algorithms. It involved the application of real-world scenarios of machine learning in biomass energy production with case I: Optimal Harvesting Time Prediction for Energy Crops, case II: Optimization of Control Parameters in Anaerobic Digestion System, and Case III: Predictive Maintenance of Biomass Combustion System. The study revealed that the implementation of ML in biorefineries has benefited in enhancing their performance, particularly in process efficiency, energy yield, and resource utilization in cases I and II. In case II, it was found to improve the overall performance of the bioenergy systems, including the optimization of key characteristics, adaptations to changing conditions, etc. (Onu et al. 2024). An AI-based control system that enhanced the reliability of feedstock preprocessing equipment by over 50% was implemented by the Idaho National Laboratory’s bioenergy program (U.S. Department of Energy 2017). It was noted that the system achieved a highly efficient operation, maintaining 97% reliability at 90% capacity. This represents a significant advancement in biorefinery operations, as such performance levels are rarely achieved during start-up, where operating capacity typically remains below 20% (Clauser et al. 2022). In a study by Azad et al. (2025), orthogonal experimental design (OED) was integrated with ML to refine NaOH-catalyzed Triton-X 100 pretreatment and enzymatic hydrolysis in sugarcane bagasse. Notably, the combined OED-ML approach effectively maximized cellulose and hemicellulose recoveries, along with glucose and xylose yields, while also minimizing the relative errors in the validation of the experiments. Feature importance analysis identified that time, temperature, and solid loading were the key factors involved in the fractionation of the biomass (sugarcane bagasse), with substrate loading having the greatest impact on sugar production. Economically, this strategy could boost bio-based sugar production profits by over $300 million by 2025, with continued growth expected by 2030, while reducing sugarcane bagasse waste by around 45% (Azad et al. 2025).

A study by Löfgren et al. (2022) optimized the AquaSolv (AqSO) biorefinery for lignin using Bayesian optimization and ML frameworks. The AqSO was developed as a green biorefinery for the integrated utilization of all the components in the biomass, particularly emphasizing the lignin-containing streams. This integrated tool was found to help in relating the biorefinery conditions, like the severity of the pretreatment reactions and temperature, to the output conditions, such as the structural features of lignin (characterized using 2D NMR). Bayesian Optimization (BO) was employed to construct surrogate models for predicting lignin yield and structural moieties (via 2D NMR) from processing parameters such as temperature and P-factor. Using lignin depolymerization to platform chemicals as a case study, a Pareto front analysis was performed to identify conditions that balance high yield with high β-O-4 content. Under these conditions, the resource-efficient extraction of the β-O-4-rich lignin was found to be possible (Löfgren et al. 2022). A study by Prioux et al. (2022) presented a five-step, generic, and practical framework for applying ML in sustainability within the Big Data context and enhancing traditional LCA by its integration in data science and AI algorithms. Public scientific literature was used as a database and analyzed using multidimensional scaling (MDS) to cluster the environmental impacts. The case study involved the comparison of biomass pretreatment processes for glucose production. The study revealed information regarding the land-use and chemical pollution clusters. While cost- and time-efficient, the approach is limited by low-TRL data, lack of scale-up considerations, and insufficient data quality for the new technological processes (Prioux et al. 2022).

### An overview of the use of ANN-based modeling in biorefineries

The demand for using computing tools has tremendously increased for data evaluations in the bioprocessing sector in recent years. The main objective of these tools is to suggest optimal, or rather the best, parameters to succeed in maximal yields. Using such tools also helps reduce experimental costs by reducing time and manpower while providing robust solutions. Currently, different types of modelling tools are in use, like the ANN, wavelet transform, fuzzy logic, genetic algorithm, etc. Among these, the ANN is widely used in the bioprocessing sector (Bhange et al. 2019). ANN and ANFIS (Adaptive neuro-fuzzy inference systems) are known to show high predictability for different pretreatment processes, including but not limited to organosolv, microwave-assisted, ultrasound-assisted pretreatments, and thermal processes like gasification, and pyrolysis, enzymatic hydrolysis, fermentation processes, etc. The tools are also known to be efficient in predicting the elemental composition and thermal characteristics of biomass by using only compositional data as input information. They have also shown excellent results in the attainment of suitable operational conditions for the efficient production of different bioproducts, including bioethanol, biogas, organic acids, lignin, and enzymes (Pradhan et al. 2022). ANN has input, hidden, and output layers, which are connected with the hidden layers of neurons or nodes (Tantoco et al. 2023). Another ANN model, gradient boosting machines, was used to predict and optimize methane yields during the anaerobic digestion of LCB (Santhosh et al. 2024).

A study by Luo et al. (2021) used ANN models for the prediction of phenolic and glucose content from the dilute inorganic pretreatment of LCB (corn stover) (Luo et al. 2021). ANN is a simplified but popular mathematical tool that is almost similar to the human central nervous system due to its characteristics. It contains an interconnected network of neurons that carries our data processing and simulations. The ANN analyses and designs the relation between the operating/ independent parameter and the response/dependent parameter of the complex system. It also includes storing data and recognizing patterns, and it learns from historical data and artificial intelligence. The ANN has been used in various fields like biomaterial hydrolysis, succession of phytoplankton, biodiesel production, COD removal, distillery wastewater treatment, pharmaceutical research, etc. (Bhange et al. 2019).

The ANN model with 4 inputs and 4 hidden neurons, which was calibrated based on the dataset on pine, was found to be accurate and robust enough in order to predict the aspect ratio of the micro/nanofiber materials from cellulose sources like softwood and hardwood species like spruce, eucalyptus, and aspen, with a correlation value of 0.84. These results reveal the usefulness of the neural network modelling strategy to predict the aspect ratio of the (ligno)cellulose micro/nanofibers in light of operation variables, which are easy to measure, and the initial fiber characteristics. This also makes it very attractive for application in industries to control product quality (Santos et al. 2022). ANN models were also used to predict the delignification in sugarcane bagasse via alkaline hydrogen peroxide (H_2_O_2_) pretreatment. The temperature of the pretreatment varied from 25 to 45 °C, and the concentration was 1.5 to 7.5% (v/v). The analytical results were obtained by Fourier Transform Infrared (FTIR) analysis and were used for the ANN training and testing steps, for the development of ANN models. From the experiments, the pretreatment at 25 °C with 4.5% (v/v) H_2_O_2_ was found to be the best for delignification, oxidizing 54% of total lignin. The coefficient, R^2^ of the model was close to 1, indicating good agreement of the theoretical and actual data (Valim et al. 2017).

The prediction of bioethanol production from LCB was also studied using two algorithm types, the artificial neural network (ANN) and the random forest algorithm (RF). The data used in the study were the results of bioethanol production from different biomass, such as buckwheat straw and its biomass, and a mixture of different biomass, as a result of ionic liquid pretreatment. The input value in the model consisted of the different ionic liquids used (imidazolium and ammonium), preparation of the enzyme, dosage of enzyme, pretreatment time and temperature, and the type of yeast used for alcoholic fermentation. As for the output value, it was the ethanol concentration. In the ANN models, a multilayer perceptron (MLP) was used. The study generated two hybrid models that were efficient for the initial screening of plants without having to perform lengthy research related to the ionic liquid pretreatment and further hydrolysis. The models require only the LCB composition data to determine their efficiency in the ionic liquid pretreatments (Smuga-kogut et al. 2021). Similarly, a study was performed to determine the efficacy of ionic liquid pretreatment on LCB using ANN and random forest (RF) regression. From the study, it was observed that the temperature and time of the pretreatment have a major role as predictors, except for hemicellulose recovery (Mahanty et al. 2024). Using ANN, Bhange et al. (2019) studied the pretreatment process of garden biomass and compared the results obtained with the response surface methodology. It was found that the ANN was an effective tool for modelling the experimental data of cellulose and lignin degradation from the pretreated LCB, and it also showed a good match with experimental data compared to the RSM (Bhange et al. 2019).

A study by Parkhey et al. (2020) also compared the ANN and RSM approaches to analyze the effectiveness of each method in the optimization of pretreatment and saccharification efficiency in rice straw. The pretreatment under study was alkali-based microwave-assisted alkali pretreatment of rice straw. It was observed that the ANN model and RSM model both obtained R^2^ values near 1, which shows their significance as models for prediction. However, due to the lower percentage of error in the ANN models for cellulose recovery and saccharification efficiency, they were found to be authoritative over RSM for exemplifying the non-linear behaviors of the system (Parkhey et al. 2020). Aruwajoye et al. (2022) found that the decision tree and the ANN models predicted the best fermentable sugar concentration in the pretreated waste-cassava peels with an accuracy of 77% and 82%. The ANN models also predicted the Combined severity factor with an accuracy of 78%, making it a better prediction model (Aruwajoye et al. 2022). ANN model has also been observed as the superior model to RSM, due to its higher prediction of the yield in a study on Ammonia-based pretreatment of corn stover (Hundie 2021). A fuzzy c-means (FCM)-clustered Adaptive neuro-fuzzy inference systems (ANFIS) and artificial neural network (ANN) model were used to model the biogas and methane yield from the anaerobic digestion of the shells of *Arachis hypogea*. The FCM-ANFIS models were found to be more efficient (R^2^ = 0.9850) than the ANN model (R^2^ = 0.9496), and hence the former can be applied to similar studies (Olatunji et al. 2023).

Different variants of the ANNs have also been used to model biogas production. They include feed-forward neural networks (FFNNs), recurrent neural networks (RNNs), and nonlinear autoregressive exogenous (NARX) networks (Alrowais et al. 2024). Coffee wastes have a porous and cellulosic structure, which aids them in having high acoustic and heat insulation properties in the building materials to which they are applied. ANN-based modelling was used to analyze the thermal properties of the insulation plasters using the data from the coffee waste-based insulators prepared from untreated and pretreated coffee waste with microwave and ultrasound pretreatments (Yildiz and Kalem 2023).

Kukić et al. (2023) studied an ANN model with input values as initial Cu^2+^ concentration, time, and particle size for predicting the adsorption capacity of the biosorbents generated from sugar beet shreds. The model was fit with an R^2^ value of 0.998. The model was found to be a good interpolation tool, whilst providing a good prediction of copper adsorption by the sugar beet shreds (Kukić et al. 2023). ANN modeling has also been used to predict the biogas prediction curves from different LCB mixtures of cattle dung with bamboo dust, sugar cane bagasse, and sawdust (Ghatak and Ghatak 2018). ANN has also been used to model the anaerobic codigestion of alkali-treated corn stover and poultry manure to maximize biogas production (Aklilu and Waday 2023).

The application of ML on Life Cycle Assessment (LCA) has also been reported by Long and Liu (2023). The availability and accuracy of the data play a huge role in the development of an LCA model since they directly impact the effectiveness as well as reliability of the model. Integration of ML can potentially improve the data quality, thus leading to an enhanced LCA performance (Long and Liu 2023). Long and Liu (2023) used different ML algorithms, like the ANN, random forest, and extreme gradient boosting, to predict the ethanol yield and NaOH consumption as key life cycle inventories for the bioethanol refineries. The prediction models were found to have high accuracy rates for ethanol yield (~ 0.85) and NaOH consumption (> 0.8). They also applied different algorithms like genetic algorithms, particle swarm optimization, and simulated annealing for the optimization of feedstock characteristics and conditions leading to a maximal ethanol yield, which showed an improvement of 18%. They also integrated the prediction and optimization algorithms with the LCA model, GREET1, to enhance ethanol yield and control the greenhouse gas emissions from the target feedstocks (Long and Liu 2023). RSM and ANN were employed by Tantoco et al. (2023) to optimize and model the enzymatic hydrolysis and fermentation conditions of polyhydroxybutyrate (PHB) production from pretreated corn stover. The ANN models in this case, too, showed higher accuracies (Tantoco et al. 2023).

#### Other models and ML combinations in biorefineries

Vinitha et al. (2024) used an Artificial Intelligence Decision-Making System (AIDMS) to optimize the bioethanol yields by making use of the given biomass characteristics (composition of cellulose, hemicellulose, and lignin) and process parameters like the acid concentration, time, and temperature of fermentation and saccharification. Different biomass was used in the study, including wheat stalk, cotton stalk, banana plant waste, rice straw, corn cob, potato peel waste, olive tree, and coconut shells. The AIDMS model also showed a 94% accuracy when compared to the experimental data for ethanol production (Vinitha et al. 2024).

The application of AI and ML techniques has improved the information content regarding the spectral data. The analysis of FTIR spectroscopic data of different LCB biomass as a rapid and non-invasive analysis of cellulose and lignin was performed by Pancholi et al. (2023). For the classification of the differences in the cellulose and lignin data of different species, they used linear discriminant analysis, decision trees, and random forest algorithms. The best classification accuracy was observed in the random forest algorithm with an accuracy score of 0.75, which also showed the least mismatches between the true and the predicted values. The Convolutional Neural Network (CNN) modelling with Bayesian regularization training algorithm using the FTIR absorbance parameters showed a good representation of LCB data with a root mean square error of ~ 0.11 (Pancholi et al. 2023).

A study by Konishi (2020) used a Deep Neural Network (DNN) to estimate the cell growth and ethanol production from the different hydrolysates using the volatile composition of the LCB. The significant compositions were estimated using the Asymmetric Autoencoder-decoder (AAE). A six-layer DNN estimated the overall growth of yeast and ethanol fermentation with good accuracy, with the losses in learning and validation as 0.033 and 0.507, respectively. In contrast, AAE decoded the volatile compositions and revealed significant features of the hydrolysates for bioethanol production, which are usually lost during the conventional approaches. The study reported that the approach integrating DNN and AAE was useful in the bioconversion of raw materials into bioethanol and other bioproducts (Konishi 2020). A study by Kovačić et al. (2024) studied different machine learning models to evaluate and predict the role of thermal pretreatment on acid-insoluble detergent lignin (AIDL) from LCB, including maize stover, sunflower stalk, soybean straw, etc. The different machine learning models employed to predict the AIDL from the LCB sources in the study included Random Forest (RF), Support Vector Machine (SVM), and Extreme Gradient Boosting (XGB). The XGB model was found to result in the highest accuracy for all the biomass, with a coefficient of determination (R^2^) in the range of 0.756–0.980. The relative variable importance from the study strongly suggests the dominance of temperature on the rate of delignification for all the biomass samples, as compared to the time of pretreatment. Thus, the effectiveness of using machine learning was proved by the study, as it helped in the optimization of the LCB pretreatment, culminating in a higher efficiency for the lignin destabilization approach (Kovačić et al. 2024).

Pushpa et al. (2023) used ML to study the variation of LCB composition between 18 rice straw samples. They developed rapid, cost-efficient, non-destructive, and less chemical-intensive machine learning models trained using FTIR spectra to quantify the LCB composition. ANN and partial least squares regression (PLSR) models were developed using the augmented spectroscopic dataset (fingerprint region of 1800 to 800 cm^− 1^) of both processed and unprocessed rice straw samples. It was observed that the ANN performed better with a coefficient of determination of 0.99 for cellulose, and ~ 0.98 for hemicellulose and lignin determination. These models could also be used in the rapid quantification of the LCB composition of rice straw, for its selection as feedstock in biorefineries (Pushpa et al. 2023). Ezzatzadegan et al. (2021) used a combined machine learning model based on the ANFIS and particle swarm optimization (PSO) to predict the production of bioethanol in biomass fermentation of oil palm trunk sap. The ANFIS model was found to be efficient for the prediction of bioethanol concentrations during the fermentation processes. The pretreatment parameters for the maximal bioethanol production were also optimised at 27.34 °C, 4.54 pH, and 16.18 h, while 1.3588 g/L sugar remained (Ezzatzadegan et al. 2021). Hybrid models like PSO-ANN, integrating particle swarm optimization (PSO) with ANN, were also designed for the estimation of glucose and xylose yields for the microwave-assisted pretreatment and enzymatic hydrolysis of the LCB (Ethaib et al. 2018). Zaied et al. (2023) used ANN coupled with particle swarm optimization (ANN-PSO) for the prediction and optimization of biogas production from the co-digestion of palm oil mill effluent in a solar bioreactor (Zaied et al. 2023).

The algorithm eXtreme Gradient Boosting was applied to predict the bio-oil yield from the solvothermal liquefaction of lignocellulosic biowaste, and the model was found to be efficient, with a prediction accuracy of (R^2^ = 94.4%) (Djandja et al. 2023). An algorithm using a stochastic gradient boosting (SGB) decision tree, another AI approach, was used to study the properties of cellulose-rich materials resulting from ionic liquid pretreatments. Using 23 different variables, they effectively determined the predictability of the output factors, including the cellulose enrichment factor and solid recovery, with R^2^ of 0.999 and 0.994, respectively (Gu et al. 2021b). Nirmal et al. (2023) also used ANN-NSGA-II-based optimization involving ANN and Non-Dominated Sorting Genetic Algorithm II to configure the optimal parameters for maximized bioethanol extractions (Nirmal et al. 2023).

## Future directions, opportunities, and applications of ML in future biorefineries

Although the direction of biorefineries is fast flourishing with many technological updates nd new product inventions, the scalability of the processes still requires further research and observations. The new products and procedures from the biorefineries are still in early technical readiness levels (~ TRL 1–6), often in the range of early laboratory validation to pilot-scale demonstration. This indicates that, while research has shown promising technical feasibility, significant efforts are still required to advance these innovations toward commercial maturity. Future research should therefore focus on optimizing process efficiency, improving product quality and consistency, validating scalability under real-world operating conditions, and conducting integrated techno-economic and environmental assessments. Collaborative industry-academia partnerships, coupled with supportive policy frameworks and investment in demonstration-scale facilities, will be critical to accelerating the transition of these technologies from experimental stages to competitive market-ready solutions. The commercialization of the LCB biorefineries and bioprocesses depends as much on institutional and policy support as on scientific progress. Clear national and regional policy frameworks are required to set targets, reduce market uncertainty, and create demand pull to lower commercial risk for developers and investors. Global and regional bioeconomy reports (IEA Bioenergy, Global Bioeconomy Policy reports) show that the lack of investments, difficulty in financing scale-ups, fluctuating biomass prices, lack of long-term stabilized policies and regulations, etc., affect the proper scale-up and working of the biorefineries (Annavelink et al., 2022). Hence, the implementation of proper policy frameworks and governance policy incentives will help establish properly functioning biorefineries.

Leveraging ML can optimize conversion processes, enhance resource efficiency, and also support the shift toward a more sustainable energy sector. Impediments like data availability, model transparency, and scalability need to be overcome to further strengthen the role of ML in biorefineries. Strong collaboration among researchers, industry, and policymakers will be key to ensuring effective implementation and broad adoption of ML in biomass energy systems. The insights gained here can help drive biomass energy toward greater efficiency, sustainability, and environmental benefits (Onu et al. 2024). ML can also be used for LCA, TEA, kinetics studies, etc. (Akinpelu et al. 2023). Huntington et al. (2023) demonstrated that the ML and decision-tree algorithms can effectively enhance conventional process simulations, enabling faster output prediction and iterative evaluation of potential equipment configurations. The surrogate models designed using the ML approaches based on conventional process simulation models like the TEA and LCA can be used as an effective tool for cost approximation, process simulations, etc. (Huntington et al. 2023). Karka et al. (2022) used ML-driven frameworks for the estimation of LCA metrics for the bio-based and biorefinery processes during their early stages of development. By combining ANNs and classification trees, environmental impacts of the biorefinery systems were predicted using minimal inputs such as molecular descriptors, feedstock traits, and process parameters. This ex-ante approach not only enables early identification of environmental hotspots but also helps in guiding sustainable design for low-TRL biorefineries. (Karka et al. 2022).

Although most ML models used in the LCB biorefineries achieve high predictive accuracy (R² >0.85), the comparison among them remains difficult, with no universally optimal model. ANN is the most widely applied method, followed by ANFIS, due to their nonlinear and adaptive capabilities for large datasets. Ongoing research continues to refine hybrid algorithms for better performance. However, challenges like model opacity (“black box” issue), discrimination and bias, the requirement for specialized knowledge, etc., exist. Effective application of ML in biorefineries demands understanding ML’s social, economic, and environmental impacts, assembling strong interdisciplinary teams, and selecting process-specific methods to gain deep insights into critical variables (Tušek et al. 2024). An illustrative case study by Shah et al. (2025) also demonstrated the utility and current dominance of the AI framework for prediction and optimization of biorefineries in the circular bioeconomy context. Critical gaps were found to exist in the use of AI, particularly in its underutilization for discovery and design functions. (Shah et al. 2025).

The future of biomass pretreatment lies in the development of smart biorefineries where the development of new production strategies aligns with the AI-based models involving ML and automation technologies, converging to create highly efficient and self-regulating systems. Digital twins, virtual replicas of biorefinery units powered by ML algorithms, could simulate various pretreatment scenarios, allowing operators to test and optimize strategies without physical experimentation. Moreover, the integration of ML with life cycle assessment (LCA) tools can also help evaluate the environmental and economic implications of different pretreatment strategies. This will enable more sustainable decision-making in the design and operation of biorefineries. Although vast research has been performed focusing on the impact of ML in bioethanol and other biofuel biorefineries, the applications of ML in advanced product design are very limited. Future studies on the integration of these advancements and ML may aid in overcoming the bottlenecks in their production, while simultaneously reducing the cost and time of their optimization.

ML has thus become an indispensable tool for the development of futuristic biorefineries. Its capabilities in modeling complex systems, optimizing process parameters, and enabling real-time control offer a significant edge over traditional methods. Although challenges in the process remain, particularly in terms of data availability and model interpretability, they may be addressed through ongoing research advancements in the area of computational tools, sensor technologies, and interdisciplinary collaboration. As a result, further evolution of ML is poised to play a central role in the transition toward smarter, more efficient, and more sustainable biorefineries.

## Conclusion

Lignocellulosic biomass pretreatment remains a cornerstone of modern biorefinery design, enabling the valorization of renewable feedstocks into high-value products. With growing interest in sustainable alternatives to petrochemicals, the focus of biorefineries is rapidly shifting beyond biofuels toward the production of advanced biomaterials, including bioplastics, biocomposites, and hydrogels. These bio-derived materials hold immense potential across the biomedical and industrial sectors, making efficient pretreatment methods more crucial than ever.

In parallel, the integration of machine learning (ML) and artificial intelligence (AI) into biorefinery workflows is revolutionizing process optimization. ML algorithms can model complex relationships between biomass composition, pre-treatment parameters, and product yields, enabling predictive control and faster optimization. Techniques such as Artificial Neural Networks (ANNs), RFs, etc., are already being employed to forecast enzymatic saccharification efficiency, minimize inhibitor formation, and improve reactor design. Going forward, the convergence of green chemistry, material innovation, and data-driven process engineering will define the next generation of lignocellulosic biorefineries. A multidisciplinary approach that leverages biotechnology, materials science, and AI is key to unlocking the full potential of biomass as a platform for the circular bioeconomy.

## Data Availability

Not applicable.

## Abbreviations

LCB: Lignocellulosic Biomass
SDG: Sustainable Development Goals
WRF: White Rot Fungi
SCF: Supercritical Fluids
IL: Ionic Liquid
DES: Deep Eutectic Solvent
3D: 3-Dimensional
NCC: Nanocrystalline Cellulose
PLA: Poly Lactic Acid
CNC: Crystalline Nanocellulose
CNF: Cellulose Nanofibers
PVA: Polyvinyl Alcohol
PCL: Polycaprolactone
AC: Activated Carbon
LCNF: Lignin-Containing Cellulose Nanofiber
CF: Cellulosic Fibers
ANC: Nanocarbon
LNS: Lignin Nanosphere
CQD: Carbon Quantum Dots
LIB: Lithium-Ion Batteries
PHA: Polyhydroxyalkanoate
WPC: Wood-Polymer Composites
PHBV: Poly(3-Hydroxybutyrate-Co-Valerate)
EDLC: Electrolytic Double-Layer Capacitors
MFC: Microbial Fuel Cells
SC: Supercapacitors
XOS: Xylo-Oligosaccharides
POS: Pecto-Oligosaccharides
COS: Cello-Oligosaccharides
LDPE: Low-Density Polyethylene
AI: Artificial Intelligence
ML: Machine Learning
ANN: Artificial Neural Networks
RF: Random Forest
RSM: Response Surface Methodology
FT-IR: Fourier Transform Infrared
MLP: Multilayer Perceptron
FCM: Fuzzy C-Means
ANFIS: Adaptive Neuro-Fuzzy Inference Systems
RNN: Recurrent Neural Network
NARX: Nonlinear Autoregressive Exogenous
FFNN: Feed-Forward Neural Network
LCA: Life Cycle Assessment
AIDMS: Artificial Intelligence Decision-Making System
DNN: Deep Neural Network
AIDL: Acid-Insoluble Detergent Lignin
SVM: Support Vector Machine
XGB: Extreme Gradient Boosting
PLSR: Partial Least Squares Regression
PSO: Particle Swarm Optimization
SGB: Stochastic Gradient Boosting
